# Deficiency of lncRNA SNHG12 impairs ischemic limb neovascularization by altering an endothelial cell cycle pathway

**DOI:** 10.1172/jci.insight.150761

**Published:** 2022-01-11

**Authors:** David A. Gross, Henry S. Cheng, Rulin Zhuang, Michael G. McCoy, Daniel Pérez-Cremades, Zachary Salyers, A.K.M. Khyrul Wara, Stefan Haemmig, Terence E. Ryan, Mark W. Feinberg

**Affiliations:** 1 Department of Medicine, Cardiovascular Division, Brigham and Women’s Hospital, Harvard Medical School, Boston, Massachusetts, USA.; 2 Department of Cardiovascular Surgery, Shanghai East Hospital, Tongji University School of Medicine, Shanghai, China.; 3 Department of Physiology, University of Valencia and INCLIVA Biomedical Research Institute, Valencia, Spain.; 4 Department of Applied Physiology and Kinesiology, University of Florida, Gainesville, Florida, USA.

**Keywords:** Angiogenesis, Cardiology, Cardiovascular disease, Endothelial cells, Noncoding RNAs

## Abstract

*SNHG12*, a long noncoding RNA (lncRNA) dysregulated in atherosclerosis, is known to be a key regulator of vascular senescence in endothelial cells (ECs). However, its role in angiogenesis and peripheral artery disease has not been elucidated. Hind-limb ischemia studies using femoral artery ligation (FAL) in mice showed that *SNHG12* expression falls readily in the acute phase of the response to limb ischemia in gastrocnemius muscle and recovers to normal when blood flow recovery is restored to ischemic muscle, indicating that it likely plays a role in the angiogenic response to ischemia. Gain- and loss-of-function studies demonstrated that *SNHG12* regulated angiogenesis — *SNHG12* deficiency reduced cell proliferation, migration, and endothelial sprouting, whereas overexpression promoted these angiogenic functions. We identified *SNHG12* binding partners by proteomics that may contribute to its role in angiogenesis, including IGF-2 mRNA–binding protein 3 (IGF2BP3, also known as IMP3). RNA-Seq profiling of *SNHG12*-deficient ECs showed effects on angiogenesis pathways and identified a strong effect on cell cycle regulation, which may be modulated by IMP3. Knockdown of *SNHG12* in mice undergoing FAL using injected gapmeRs) decreased angiogenesis, an effect that was more pronounced in a model of insulin-resistant *db/db* mice. RNA-Seq profiling of the EC and non-EC compartments in these mice revealed a likely role of *SNHG12* knockdown on Wnt, Notch, and angiopoietin signaling pathways. Together, these findings indicate that *SNHG12* plays an important role in the angiogenic EC response to ischemia.

## Graphical abstract

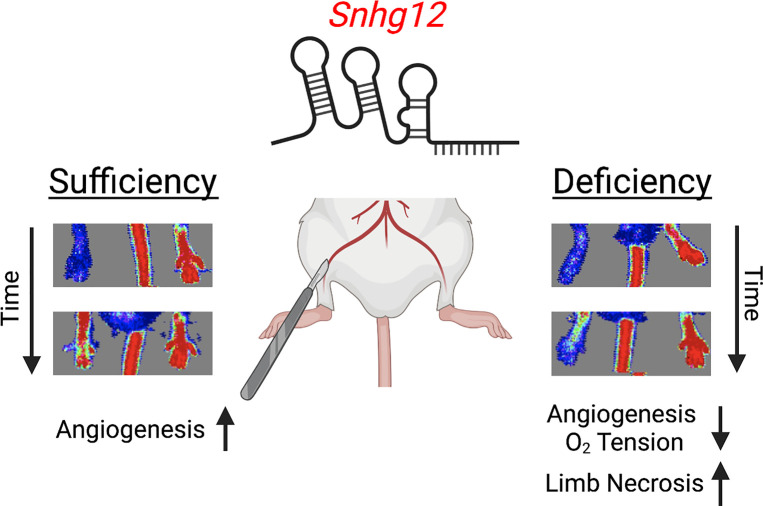

## Introduction

Peripheral artery disease (PAD) is defined by impaired blood flow to the lower extremities and is estimated to affect over 200 million people worldwide (1). Major risk factors for PAD include smoking, diabetes, age, hypertension, and hyperlipidemia, and it is known that diabetes confers adverse prognosis for patients with PAD (2). Critical limb ischemia (CLI) defines a subset of these patients who have ischemic rest pain often associated with impaired wound healing, ulceration, and infection and increased risk of lower extremity amputation and cardiovascular death (2, 3). PAD and CLI are defined by impaired angiogenic responses, which are exaggerated under diabetic conditions. Many growth factor therapies have been tried for PAD in order to improve angiogenesis, but none have resulted in successful outcomes in clinical trials, likely because of defective downstream signaling pathways, which has led to the rise of the term “angiogenic resistance” (4–8). This is plausible given that PAD patients exhibit higher expression of proangiogenic signaling pathway ligands such as VEGF and angiopoietin-2 but maintain impaired angiogenic responses to ischemia (7).

Long noncoding RNAs (lncRNAs) are recognized as important regulators of transcription, gene expression, protein turnover, and protein function (9–11). Several lncRNAs have been identified as important regulators of angiogenesis, including MALAT-1, MEG3, and ANRIL (12–17). Paradigms exist for in *cis* and in *trans* action of lncRNAs in the nucleus, affecting RNA splicing, protein scaffolding, and enhancer-like functions (13). Despite the burgeoning field of lncRNA influence over angiogenesis, little is known about the role of lncRNAs in vascular senescence in this context (14, 18–20).

Recently, our laboratory identified the evolutionarily conserved lncRNA small nucleolar host gene 12 (*SNHG12*) as being significantly decreased in aortic vascular endothelium during atherosclerotic lesion progression in mice. LncRNA pull-down studies combined with liquid chromatography–tandem mass spectrometry identified DNA-dependent protein kinase (DNAPK) as an *SNHG12*-interacting protein. Subsequent studies showed that *SNHG12* knockdown reduced the interaction of DNAPK with its binding partners, Ku70 and Ku80, that bind to DNA double-strand breaks. Carotid arteries from pigs on high-cholesterol diet and diseased human carotid arteries showed that *SNHG12* expression was abrogated according to lesion progression (21).

We hypothesized that the lncRNA *SNHG12* may play an important role in angiogenesis and sought to define this further in angiogenic studies in vitro and in vivo. We show that abrogation of *SNHG12* expression decreases the angiogenic response and identify several novel *SNHG12-*interacting proteins. Knockdown of *SNHG12* in mice undergoing femoral artery ligation (FAL) as a model for limb ischemia shows that there are decreased arterial number and arterial size, suggesting decreased angiogenesis in vivo. RNA-Seq transcriptional analysis suggests that there is defective signaling in various angiogenic pathways that may mediate this phenotype, providing new insight and the foundation for future studies on the importance of *SNHG12* in angiogenesis.

## Results

The mechanisms underlying CLI and tissue injury in subjects with diabetes are poorly understood but are thought to involve impaired angiogenesis, inflammation, and vascular and skeletal muscle dysfunction overlaid on systemic risk factors. This study sought to evaluate the role of the lncRNA *SNHG12* in the response to limb ischemia using a mouse model of acute limb ischemia.

### Expression of SNHG12 is dysregulated in PAD.

**Figure 1:**
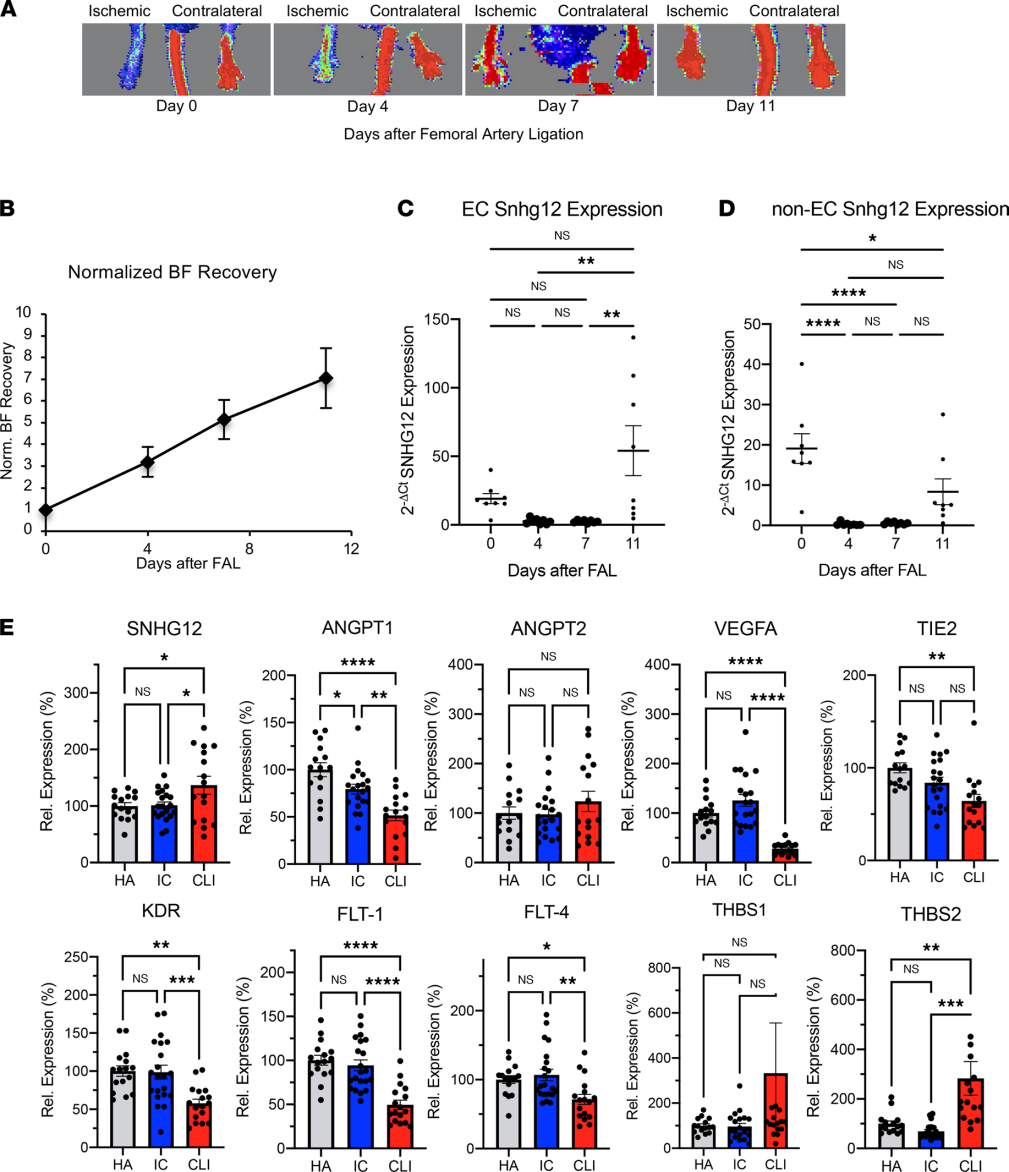
Expression of Snhg12 in murine hind-limb ischemia and SNHG12 in human PAD. Snhg12 is dysregulated in a mouse model of acute hind-limb ischemia and in human cohorts with CLI. (**A**) Anesthetized mice that underwent sham surgery (*n* = 8) or FAL (*n* = 24) were imaged by laser Doppler imaging in the supine position to evaluate blood flow (BF) in the ischemic limb versus contralateral limb on days 0, 4, 7, and 11 (representative images shown at these time points). (**B**) Laser Doppler imaging results were quantified using MoorLDI software in order to show fold increase in BF compared with the immediate postoperative ischemic BF (normalized BF recovery). (**C** and **D**) Mice were sacrificed at day 4 (*n* = 8), day 7 (*n* = 8), and day 11 (*n* = 8), and isolated gastrocnemius muscle was subjected to EC (**C**) and non-EC (**D**) isolation. RNA was isolated from respective fractions, and quantitative RT-PCR was performed for Snhg12 compared with Hprt control. (**E**) Expression of SNHG12 compared with various angiogenic genes is dysregulated in age-matched ischemic claudicant (IC) and critical limb ischemia (CLI) patients versus healthy adults (HA) from a cohort of patients aged 51–84 who underwent gastrocnemius biopsy and whole transcriptome sequencing (*n* = 15 for HA, *n* = 20 for IC, and *n* = 16 for CLI). *P < 0.05, **P < 0.01, ***P < 0.001, ****P < 0.0001 using Student’s *t* test or 1-way ANOVA with Bonferroni correction for multiple comparisons.

In order to determine the role of *SNHG12* after limb ischemia, anesthetized mice were subjected to FAL. Over the course of 2 weeks, blood flow to the ischemic paw relative to the nonischemic paw was measured serially by laser Doppler imaging (Figure 1A). Quantification and normalization of blood flow recovery to the postoperative period showed that blood flow recovery occurred over the course of 2 weeks (Figure 1B). Early after acute limb ischemia, SNHG12 levels were decreased by 78%–86% in the endothelial cell (EC) and significantly decreased by 93%–95% in non-EC compartments of gastrocnemius muscle in C57BL/6 mice at days 4 and 7, during a time that correlates with acute inflammation and initial wound healing responses involving EC interaction with inflammatory cells, fibroblasts, and smooth muscle cells. As blood flow recovery normalized, compared with days 4 and 7, SNHG12 expression at day 11 in the EC compartment was significantly increased at a time when angiogenesis plays an important role in ischemic limb tissue remodeling (Figure 1, C and D). Human SNHG12 expression has not been investigated in the context of PAD in the literature, but a recent transcriptomic evaluation of gastrocnemius muscle RNA was undertaken in non-PAD healthy adults versus patients with ischemic claudication and with CLI (22). Using this published cohort of patients, we show that SNHG12 expression was significantly increased in gastrocnemius muscle from CLI patients relative to healthy adults and ischemic claudicants, whereas levels of angiopoietin-1 (ANGPT1), VEGF-A, Tie2, and the VEGF receptors KDR, Flt-1, and Flt-4 were markedly decreased (Figure 1E). The antiangiogenic factor thrombospondin-2 (THBS2) is significantly increased in CLI patients relative to healthy adults and ischemic claudicants, and there is a nonsignificant trend toward increased expression in thrombospondin-1 (THBS1) (22).

### SNHG12 regulates angiogenesis.

**Figure 2:**
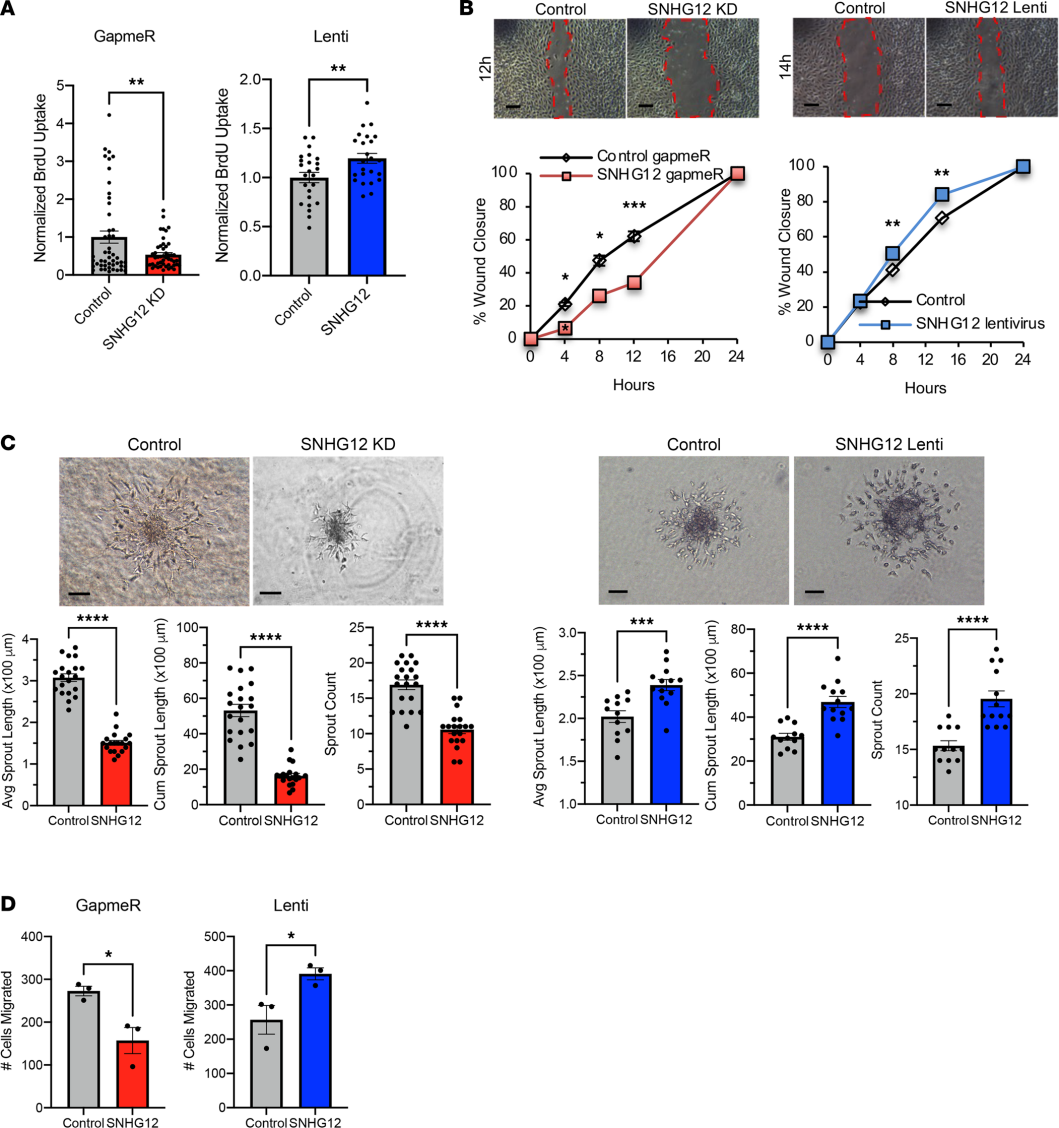
In vitro angiogenesis assays using SNHG12 gain- and loss-of-function models. Angiogenesis was assessed using BrdU uptake, endothelial wound healing (scratch) assay, EC spheroid sprouting, and Transwell migration. Knockdown of SNHG12 leads to impaired cell proliferation, migration, wound healing, and angiogenic sprouting, whereas overexpression of SNHG12 leads to increased EC proliferation, migration, wound healing, and angiogenic sprouting. (**A**) HUVECs underwent gapmeR-mediated knockdown or lentivirus-mediated overexpression of SNHG12 and were then subjected to BrdU assay 48–60 hours after transfection (*n* = 8–16 per condition). (**B**) HUVECs treated similarly with gapmeRs or lentivirus as in **A** were plated at 25,000 cells per well in scratch assay plate wells, and once cells were confluent, the divider between wells was removed, allowing cells to close the “wound” or “scratch.” Cells were imaged at 0, 4, 8, and 12 or 14 hours and 24 hours after divider removal, and percentage wound closure for each condition was measured in comparison with time point 0 (*n* = 6–8 per condition; scale bars: 200 μm). (**C**) HUVECs treated with gapmeRs or lentivirus as in **A** or **B** were made to form spheroids by hanging droplet method, plated in methylcellulose-collagen matrix, and stimulated to sprout for 18–24 hours with 50 ng/μL VEGF at 48 hours after transfection. Cells were then imaged on an upright light microscope and measured for sprout length and sprout number using ImageJ (NIH) (*n* = 20–30 spheroids were counted per condition; scale bars: 200 μm). (**D**) HUVECs were transfected with gapmeRs or transduced with lentivirus as in **A**–**C** and were then plated in Transwell migration plates at 37,500 cells per well in serum-free and growth factor–free media and allowed to transmigrate for 16 hours toward 10% FBS in EGM2 media with standard growth factor concentrations (*n* = 3 per condition). *P < 0.05, **P < 0.01, ***P < 0.001, ****P < 0.0001 using Student’s *t* test.

SNHG12 has been shown to be protective against atherosclerosis via its role in DNA damage repair, whereas knockdown of SNHG12 exacerbated aortic atherosclerosis (21). Given the role of SNHG12 in atherosclerosis and its importance in vascular EC senescence, we hypothesized that SNHG12 may play a role in angiogenesis. In order to assess cellular proliferation, SNHG12 was knocked down using locked nucleic acid gapmeRs in HUVECs and overexpressed via transduction of SNHG12-encoding lentivirus. Indeed, silencing of SNHG12 reduced EC proliferation by 56%, whereas SNHG12 overexpression increased EC proliferation by 20% as quantified by BrdU (Figure 2A). The average percentage knockdown for SNHG12 using gapmeRs at a concentration of 25 nM in HUVECs was about 60%–70%, whereas overexpression was about 8- to 10-fold (Supplemental Figure 1; supplemental material available online with this article; https://doi.org/10.1172/jci.insight.150761DS1). We also explored whether SNHG12 played a role in cell-cell interactions as assessed by in vitro “wound healing” or scratch assays. Using transfected HUVECs that were seeded into specific wound-healing microdishes, knockdown of SNHG12 significantly impaired wound closure by 55%, whereas its overexpression accelerated wound closure by 23% at 8 hours (Figure 2B). As 3D spheroids have been shown to recapitulate angiogenic sprouting with tip and stalk cells, we used a spheroid model to assess HUVEC angiogenic sprouting potential in the context of SNHG12 knockdown or overexpression. Cells that were transfected and subsequently allowed to form spheroids (at ~1000 cells per spheroid) in methylcellulose-containing serum-free EGM2 media by the hanging droplet technique were subsequently plated into a collagen-methylcellulose matrix containing VEGF and allowed to sprout for 24 hours. SNHG12 knockdown significantly impaired the number of sprouts (by 37%), average sprout length (by 52%), and cumulative sprout length (by 69%), whereas overexpression significantly increased sprout number (by 27%), length (by 18%), and cumulative length (by 51%) (Figure 2C). In order to understand whether there was also a migration defect in HUVECs with SNHG12 knockdown or overexpression, we also used Transwell migration assays in order to assess the response of synchronized cells to a high-serum chemotactic stimulus. SNHG12 knockdown resulted in a significant migratory defect (by 42%) relative to control cells, whereas overexpression accelerated cell migration (by 54%) (Figure 2D). These results demonstrate that SNHG12 plays important roles in EC proliferation, wound closure, sprout formation, and migration.

### Knockdown of Snhg12 in vivo in C57BL/6 and BALB/c mice decreases blood flow recovery after acute limb ischemia.

**Figure 3:**
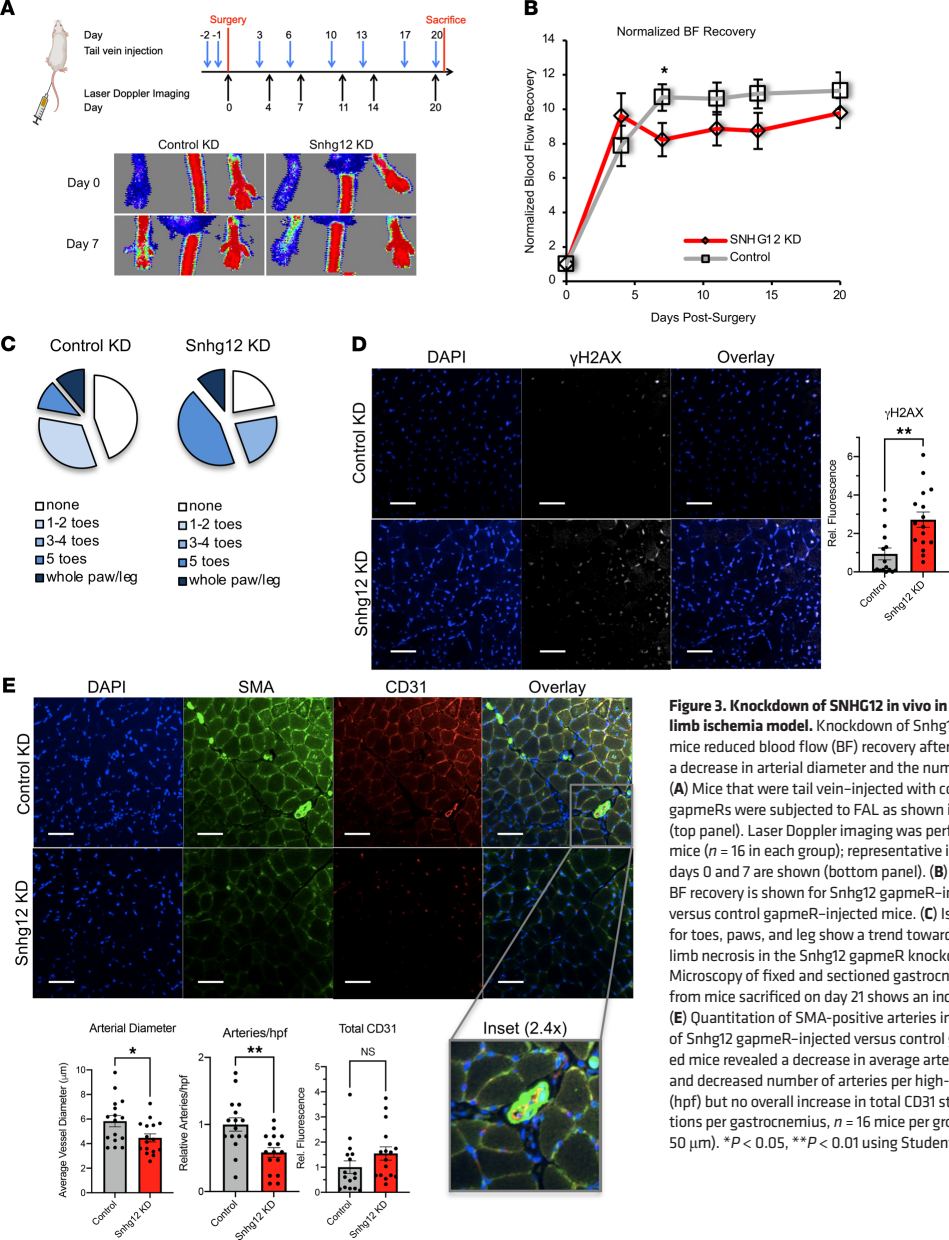
Knockdown of SNHG12 in vivo in a C57BL/6 hind-limb ischemia model. Knockdown of Snhg12 in C57BL/6 mice reduced blood flow (BF) recovery after FAL owing to a decrease in arterial diameter and the number of arteries. (**A**) Mice that were tail vein–injected with control or Snhg12 gapmeRs were subjected to FAL as shown in the schematic (top panel). Laser Doppler imaging was performed on mice (*n* = 16 in each group); representative images from days 0 and 7 are shown (bottom panel). (**B**) Normalized BF recovery is shown for Snhg12 gapmeR–injected mice versus control gapmeR–injected mice. (**C**) Ischemia scores for toes, paws, and leg show a trend toward more areas of limb necrosis in the Snhg12 gapmeR knockdown group. (**D**) Microscopy of fixed and sectioned gastrocnemius muscle from mice sacrificed on day 21 shows an increase in γH2AX. (**E**) Quantitation of SMA-positive arteries in gastrocnemius of Snhg12 gapmeR–injected versus control gapmeR–injected mice revealed a decrease in average arterial diameter and decreased number of arteries per high-power field (hpf) but no overall increase in total CD31 staining (4–5 sections per gastrocnemius, *n* = 16 mice per group; scale bars: 50 μm). *P < 0.05, **P < 0.01 using Student’s *t* test.

From in vitro angiogenic assays, a robust antiangiogenic phenotype was observed with reduced Snhg12 expression; therefore, we hypothesized that abrogation of Snhg12 expression by delivery of Snhg12 gapmeRs via tail vein injections (as demonstrated previously by our group) would lead to decreased blood flow recovery in mice undergoing FAL since they affect endothelial expression of Snhg12 and Snhg12 is not reliably detectable in plasma (21). Based on kinetic studies by Haemmig et al., mice were subjected to a 3-week protocol beginning with 2 gapmeR loading doses prior to FAL followed by biweekly gapmeR injections with serial imaging over the course of 3 weeks (Figure 3A) (21). Knockdown of Snhg12 showed a statistically significant decrease in blood flow recovery (maximally 23% at 1 week) that persisted over time in response to FAL (Figure 3B and Supplemental Figure 2). Consistent with this, there was a trend toward increased toe ischemia scores in mice with Snhg12 knockdown compared with those treated with control gapmeR. However, these studies were not powered to look for statistically significant differences in toe ischemia scores (Figure 3C). Histologic analyses of the ischemic gastrocnemius muscle revealed that knockdown of SNHG12 in C57BL/6 mice resulted in a greater percentage of small myofibers demonstrated by a leftward shift in myofiber cross-sectional area frequency histogram (Supplemental Figure 3A). Quantitative analysis of the percentage of small myofibers (cross-sectional area less than 500 μm^2^ or 1000 μm^2^) showed that knockdown of SNHG12 exacerbated the ischemic myopathy in C57BL/6 mice. Furthermore, mice that received the SNHG12 gapmeR displayed significant increases in the non-myofiber area compared with control gapmeR–treated mice (Supplemental Figure 3A), indicating an expansion of the extracellular matrix. After 3 weeks of biweekly injections with mice showing a sustained plateau of blood flow recovery at 55%–65% of baseline for Snhg12 knockdown, rather than 75%–80% of baseline, mice were sacrificed and gastrocnemius muscle was fixed, paraffin-embedded, axially sectioned, and subjected to immunofluorescence staining to assess differential angiogenic and inflammatory responses. While we were unable to show statistically different differences in Snhg12 expression at this time point (Supplemental Figure 2B), our prior studies in lesions of atherosclerotic mice showed that Snhg12 knockdown increased γH2AX, a marker of DNA double-strand breaks, and we used this as a surrogate for cumulative exposure of muscle to Snhg12 knockdown. To assess for DNA damage in ischemic gastrocnemius of Snhg12 knockdown mice, we stained for γH2AX and found a 287% increase in comparison with control-injected mice (Figure 3D). We next assessed for effects on angiogenesis as measured using CD31 staining with arterioles being delineated by the presence of smooth muscle actin (SMA). While there was no difference in total CD31 staining in whole gastrocnemius, measurement of SMA-positive vascular structures showed a significant decrease in average arterial diameter (by 23%) and number (by 42%) in ischemic limbs of Snhg12-knockdown mice (Figure 3E). Using CD45 and M1 (CCR7) or M2 (CD206) markers, we did not observe differential monocyte accumulation or macrophage polarization in ischemic limbs of Snhg12-knockdown relative to control groups (Supplemental Figure 2C).

Given the relatively subtle differences in blood flow with tail vein–injected mice and the ultimate hepatic metabolism of the gapmeRs, we hypothesized that local intramuscular gapmeR delivery would lead to a more robust model capable of demonstrating differences in hind-limb ischemia and better preserved knockdown kinetics. Furthermore, local gapmeR delivery would negate significant first-pass metabolism by the liver that occurs upon tail vein injection. Using C57BL/6 and *db/db* mice injected with control or Snhg12 gapmeRs for 2 consecutive days, we sacrificed mice on day 3 and isolated RNA from liver, PBMCs, and gastrocnemius EC and non-EC fractions. This showed that there was a significant decrease in expression of Snhg12 in the EC and non-EC fractions of gastrocnemius in C57BL/6 and *db/db* with 2 loading doses (Supplemental Figure 4).

In order to further probe blood flow recovery in response to Snhg12 depletion, we used an alternate hind-limb ischemia model and performed similar FAL experiments in 8- to 10-week-old BALB/c mice, now using intramuscular gapmeR delivery. BALB/c mice are known to have a higher propensity to require amputation after hind-limb ischemia. In order to understand the kinetics of Snhg12 expression in this mouse model, we performed FAL in BALB/c mice followed by serial laser Doppler imaging over the course of 12 days, showing that blood flow recovered to approximately 70%–80% between 7 and 11 days after FAL (Supplemental Figure 5, A–C). Snhg12 expression in gastrocnemius isolated from the ischemic limb showed a statistically significant increase at day 7 compared with day 0, demonstrating expression kinetics after FAL that differed from those in C57BL/6 mice in addition to an overall lower level of expression (Supplemental Figure 5D and Figure 1, C and D). Toe ischemia scores showed a trend toward more severe phenotype in comparison with C57BL/6 mice, consistent with what is generally observed in BALB/c mice undergoing FAL (Supplemental Figure 5E).

To add to the experiments performed in C57BL/6 mice shown in Figure 3, 8- to 10-week-old BALB/c mice were loaded with 2 consecutive daily doses of control or Snhg12 gapmeR delivered intramuscularly into ipsilateral gastrocnemius prior to FAL. After FAL, these mice subsequently received biweekly injections with serial laser Doppler imaging for 2 weeks (Supplemental Figure 6A). After 2 weeks, once a plateau of blood flow recovery was obtained in balance with the high amputation rates observed in these mice, the mice were sacrificed and gastrocnemius tissue was captured for histologic processing as performed in C57BL/6 mice (Figure 3). Blood flow recovery in BALB/c mice treated with Snhg12 gapmeR was only 35%–40%, demonstrating a sustained plateau between 1 and 2 weeks, whereas blood flow recovery in control gapmeR–treated mice returned to a plateau of 75%–80% (Supplemental Figure 6, B and C). To supplement laser Doppler imaging data, we used direct tissue oxygen measurement in bilateral mid-gastrocnemius muscles of these mice (OxyLite Pro Ltd., Oxford Optronics) (23, 24) once a plateau was reached in blood flow recovery in comparison with the day of surgery. This confirmed a severe muscle perfusion defect immediately after surgery with tissue oxygen tension less than 1 mmHg in the ligated limb, whereas by day 10 in the ligated limb, control gapmeR–injected mice had oxygen levels of 24.3 mmHg while Snhg12 gapmeR–injected mice had significantly lower oxygen levels of 6.6 mmHg (Supplemental Figure 6D). Notably, there was significantly higher baseline oxygen tension in Snhg12-knockdown ischemic limbs versus control ischemic limbs at day 0 after FAL; however, given the difference of less than 0.15 mmHg, this is unlikely to be biologically relevant. The nonligated, uninjected limbs (non-FAL limbs) had their oxygen tension measured as a control and were found to be similar between both treatment groups at day 0 and day 10 (tissue oxygen measurement of 31–33 mmHg) and similar to what is known about oxygen concentration in resting gastrocnemius muscle tissue in anesthetized mice and humans (Supplemental Figure 6D) (25–27). Ischemia scores for these mice showed a significant trend toward increased ischemia (Supplemental Figure 6E). Snhg12 expression was confirmed to be significantly reduced by 55% in the EC fraction of cells from gastrocnemius isolated from the FAL limb treated with Snhg12 gapmeR versus control, demonstrating better gapmeR kinetics than using tail vein injections (Supplemental Figure 6F). Thirty minutes before sacrifice, animals were injected with *Lycopersicon esculentum* FITC-lectin via tail vein injection in order to label perfused capillary beds as reported by others (28). Gastrocnemius was then isolated, fixed, paraffin-embedded, and stained for CD31, SMA, and DAPI. Histologic analysis of gastrocnemius isolated from the ligated unamputated limbs of BALB/c mice showed a 23%–28% reduction in average arterial diameter (measured by isolectin or CD31) and a 46% reduction in the number of arteries per high-power field without a change in total CD31 or isolectin staining (Supplemental Figure 6G).

Together, these data suggest that SNHG12 plays a role in EC angiogenesis that is not caused by increased monocyte migration or differential macrophage polarization that modulates the inflammatory phenotype in the wound healing response to ischemia. While Snhg12 expression appears to play an important role in the angiogenic response in acute limb ischemia, the mechanism by which this occurs is not solely explained by its interaction with DNAPK and its role in vascular senescence, suggesting a role in cellular proliferation, growth pathways, response to VEGF, endothelial sprouting, or the response of skeletal muscle and the EC compartment to ischemia.

### SNHG12 interacts with several proteins.

**Figure 4:**
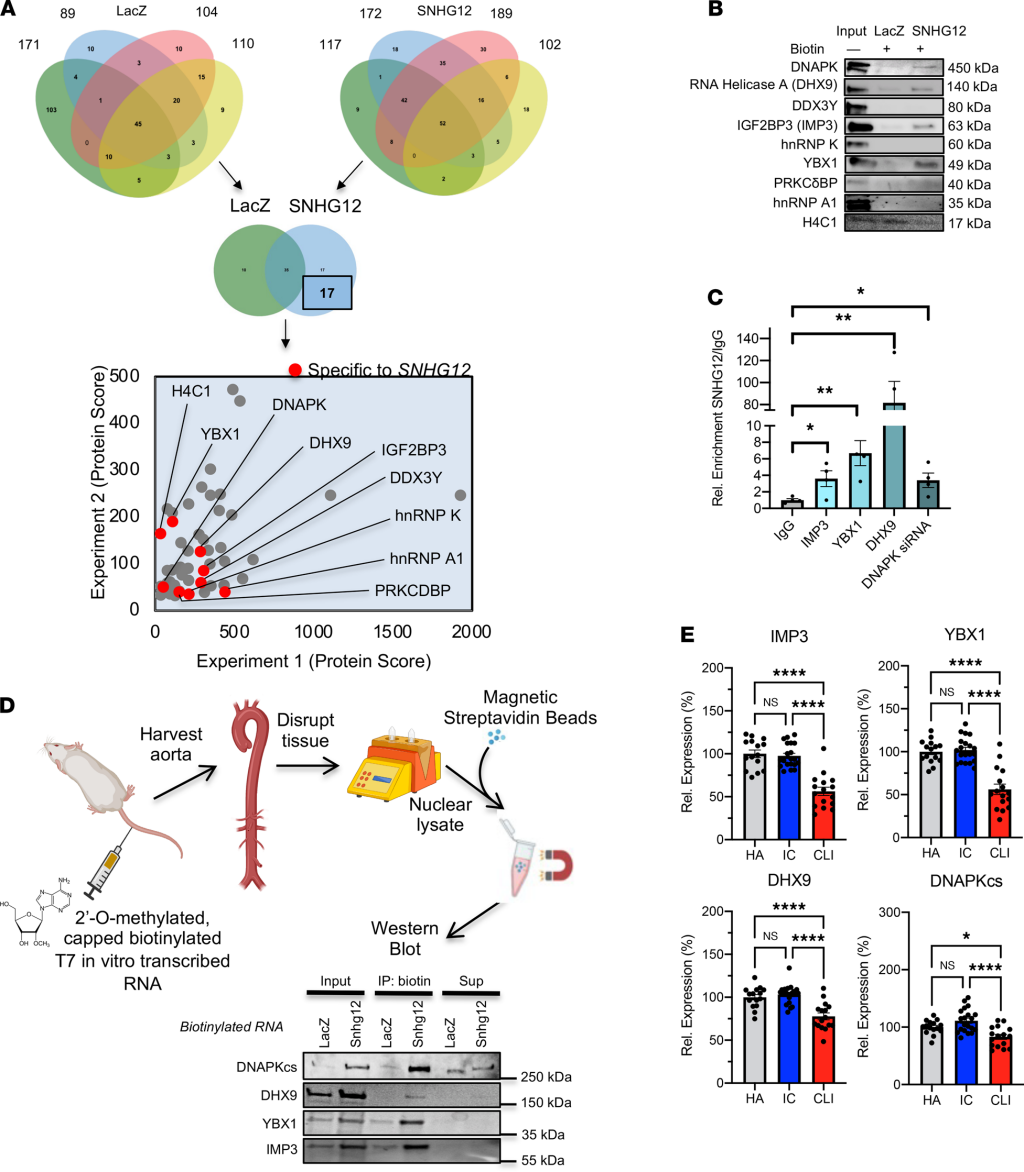
Pull-downs of SNHG12-interacting proteins. SNHG12 not only interacts with DNAPK but also interacts with several other proteins as shown in vitro and in vivo. The expression of these is decreased in gastrocnemius muscle in CLI in a human cohort. (**A**) Proteomics was performed and yielded protein hits from both biotin-labeled *LacZ* and *SNHG12* (*n* = 2 biological replicates and *n* = 2 technical replicates each). Common proteins were overlaid for each group, and those unique to SNHG12 demonstrated several unique nonribosomal proteins as shown in the protein score plot. (**B**) RNA immunoprecipitation pull-down of biotinylated in vitro–transcribed LacZ or SNHG12 RNA with HUVEC lysate shows specific enrichment of DNAPK, IGF2 mRNA–binding protein 3 (IGF2BP3, also known as IMP3), RNA helicase A (DHX9), and YBX1 (*n* = 3). (**C**) LncRNA pull-down of SNHG12 by immunoprecipitation of IMP3, YBX1, DHX9, and DNAPK from HUVEC nuclear lysate shows enrichment of SNHG12 compared with negative control (IgG) (*n* = 3). (**D**) Mice injected with in vitro–transcribed 2′-*O-*methylated, biotinylated LacZ or Snhg12 underwent aortic harvesting, tissue disruption, nuclear isolation, and subsequent immunoprecipitation using magnetic streptavidin beads, showing enrichment of DNAPK, DHX9, IMP3, and YBX1 (from aortae pooled from 4 mice in each group). (**E**) Expression levels of IMP3, YBX1, DHX9, and DNAPKcs are all decreased in CLI patients versus ischemic claudicants (IC) or healthy adults (HA) from a cohort of patients aged 51–84 who underwent gastrocnemius biopsy and whole transcriptome sequencing (*n* = 15 for HA, *n* = 20 for IC, and *n* = 16 for CLI). *P < 0.05, ****P < 0.01 using Student’s *t* test or 1-way ANOVA with Bonferroni correction.

Prior experiments in our laboratory using mass spectrometry identified the most specific interaction of biotinylated SNHG12 lncRNA with DNAPK (21), showing its importance in vascular senescence; however, these data suggested that SNHG12 may also bind several other proteins not known to play roles in vascular homeostasis (Figure 4A). Excluding cytosolic or ribosomal proteins as less likely archetypal targets of lncRNAs, we were left with several unique proteins in addition to DNAPK in decreasing order of specificity: ATP-dependent RNA helicase A (DHX9), ATP-dependent RNA helicase DDX3Y, heterogeneous nuclear riboproteins A1 isoform a and K isoform b (hnRNPs A1 and K), nuclease-sensitive element–binding protein 1 (YB1/YBX1), insulin-like growth factor 2 mRNA–binding protein 3 (IGF2BP3, also known as IMP3), and histone H4 (H4C1) (Figure 4A and Supplemental Figure 7). In order to verify these interactions, biotin-labeled, in vitro–transcribed, and folded SNHG12 lncRNA versus a negative control (LacZ RNA) was allowed to interact with HUVEC nuclear lysates and subsequently pulled down using magnetic streptavidin beads. Biotinylated SNHG12 not only specifically immunoprecipitated DNAPK compared with LacZ RNA, but also pulled down DHX9, IMP3, and YBX1. However, biotinylated SNHG12 did not pull down DDX3Y, PRKCDBP, hnRNP A1, hnRNP K, or H4C1, which were similarly not bound in the LacZ immunoprecipitate (Figure 4B). In the converse experiment, HUVEC nuclear lysates that were immunoprecipitated with IMP3, YBX1, DHX9, and DNAPK versus IgG antibodies as a negative control were able to enrich for SNHG12 (Figure 4C). This interaction was validated in vivo by intravenous injections of in vitro–transcribed, biotin-labeled, 5′-capped, and 2-*O*-methylated SNHG12 or LacZ in C57BL/6 mice. Biotin-labeled SNHG12 was able to pull DNAPK, IMP3, YBX1, and DHX9 (Figure 4D). Interestingly, the levels of these transcripts were all significantly decreased in CLI patients versus non-PAD healthy adults and ischemic claudicants (Figure 4E) (22).

**Figure 5:**
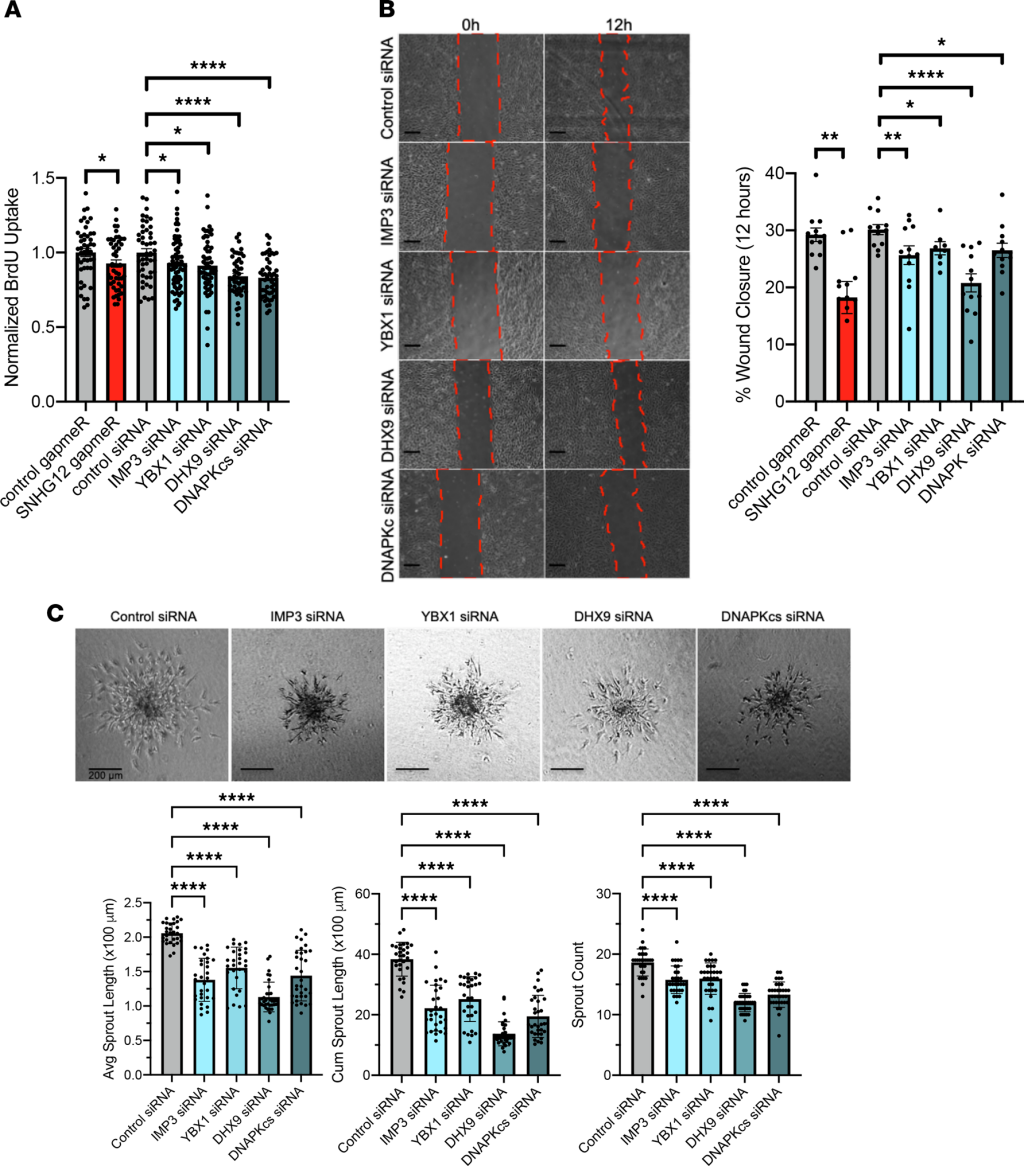
Angiogenesis functional assays of SNHG12-interacting proteins. Angiogenesis assays of SNHG12-interacting proteins revealed that their knockdown reduced cell proliferation, wound closure, and spheroid sprouting. (**A**) BrdU assays in HUVECs with siRNA-mediated knockdown against IMP3, YBX1, DHX9, or DNAPK show a decrease in cellular proliferation relative to control siRNA, similar to findings with SNHG12 gapmeR knockdown (*n* = 20–24 per condition). (**B**) Wound healing (“scratch”) assays show that siRNA-mediated knockdown of IMP3, YBX1, DHX9, or DNAPK decreases percentage wound closure relative to control siRNA knockdown (*n* = 4–6 per condition; scale bars: 200 μm). (**C**) Spheroid sprouting assays performed in HUVECs with siRNA-mediated knockdown of IMP3, YBX1, DHX9, or DNAPK show decreased sprout length, sprout count, and cumulative sprout length relative to control siRNA knockdown spheroids (*n* = 20–30 spheroids per condition; scale bars: 200 μm). *P < 0.05, **P < 0.01, ****P < 0.0001 using Student’s *t* test or 1-way ANOVA.

In order to understand the role of these proteins in angiogenesis, we sought to evaluate the consequences of siRNA-mediated knockdown of SNHG12’s interactors. Silencing RNAs targeted toward IMP3, YBX1, DHX9, and DNAPK were transfected into HUVECs, which were subsequently used for BrdU, scratch, and sprout assays. Knockdown of these proteins significantly decreased cellular proliferation (7%, 7%, 19%, and 20%) induced by IMP3, YBX1, DHX9, and DNAPK knockdown, respectively, and was similar to or greater than SNHG12 knockdown using gapmeR-mediated silencing. The severity of phenotype was likely due to the higher degree of knockdown efficiency with siRNAs relative to gapmeRs (Figure 5A and Supplemental Figures 1 and 8). Knockdown of SNHG12-interacting proteins also revealed impaired in vitro wound healing (Figure 5B). More pronounced was the impact of knockdown of IMP3, YBX1, DHX9, or DNAPK on sprout formation, which led to significantly abrogated sprout number (by 16%, 14%, 35%, and 28%, respectively), length (by 33%, 24%, 48%, and 33%, respectively), and cumulative sprout length (by 41%, 34%, 64%, and 49%, respectively) (Figure 5C).

### Snhg12 knockdown in a model of diabetes leads to impaired blood flow recovery after FAL.

**Figure 6:**
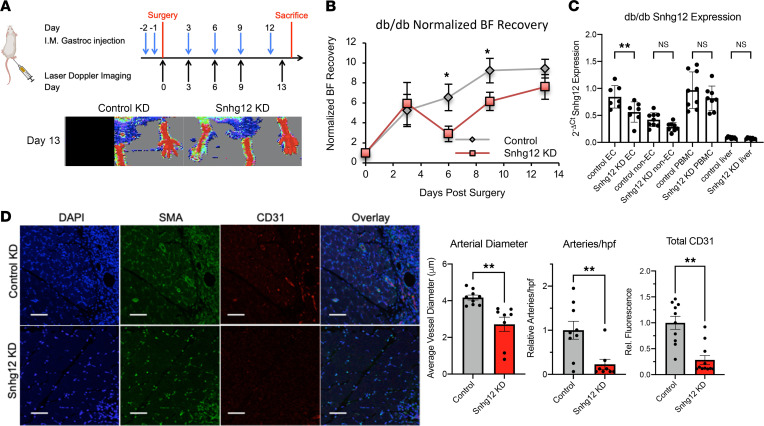
Knockdown of Snhg12 in a diabetic hind-limb ischemia model exacerbates neovascularization. Knockdown of Snhg12 in *db/db* mice results in a reduction in blood flow (BF) recovery after FAL due to a decrease in arterial diameter and the number of arteries and overall CD31 expression in muscle. (**A**) Mice were intramuscularly injected with control or Snhg12 gapmeRs into gastrocnemius muscle and subjected to FAL as shown in the schematic (top). Laser Doppler imaging was performed on mice (*n* = 8–9 in each group); representative images from days 0 and 13 are shown (bottom). (**B**) Normalized BF recovery is shown for Snhg12 gapmeR–injected mice versus control gapmeR–injected mice. (**C**) Quantitative RT-PCR expression of SNHG12 in gastrocnemius ECs and non-ECs, PBMCs, and liver of mice treated with intramuscular injection of control or Snhg12 gapmeR (*n* = 8–9 per condition). (**D**) Microscopy of fixed and sectioned gastrocnemius muscle from mice sacrificed on day 14. Quantification of SMA-positive arteries in gastrocnemius of Snhg12 gapmeR–treated mice relative to control gapmeR–treated mice revealed a decreased average arterial diameter and decreased number of arteries per high-power field (hpf) in addition to an overall decrease in total CD31 staining (20–25 fields per gastrocnemius, *n* = 8–9 mice per group; scale bars: 50 μm). *P < 0.05, **P < 0.01 using Student’s *t* test.

Given that diabetes is a major risk factor for the development of PAD (2, 6), progression of PAD, and major adverse limb events and cardiovascular events, we evaluated the role of Snhg12 silencing in a mouse model of obesity and diabetes. Commercially available *db/db* mice at 8–10 weeks of age were loaded with 2 consecutive daily doses delivered intramuscularly into gastrocnemius ipsilateral to the side of FAL and received biweekly injections with serial laser Doppler imaging for 2 weeks, until blood flow recovery had plateaued (Figure 6A). Consistent with what was observed in C57BL6 mice with tail vein–injected naked gapmeRs, Snhg12 knockdown in *db/db* mice delivered locally to the gastrocnemius muscle showed significantly reduced blood flow recovery as early as 6 days postoperatively (by 56%), and there was a trend toward increased toe, paw, and leg ischemia scores (Figure 6B and Supplemental Figure 9, A and B). The kinetics of Snhg12 expression in *db/db* mice after FAL were somewhat similar in trend to those observed in C57BL/6 mice although without significant differences observed at day 3 or day 11 (Supplemental Figure 9C). Snhg12, which was more highly expressed in the gastrocnemius EC fraction, was reduced by 30%–35%. A similar percentage knockdown was achieved in the gastrocnemius muscle non-EC fraction; however, there was no significant Snhg12 knockdown in PBMCs or liver (Figure 6C). Like in C57BL/6 mice, SNHG12 knockdown in *db/db* mice resulted in a leftward shift in myofiber cross-sectional area frequency histogram and a significant increase in the number of myofibers with cross-sectional area less than 500 μm^2^ in comparison with control knockdown mice (Supplemental Figure 3B). The percentage of non-myofiber area (extracellular matrix) was modestly increased in SNHG12-knockdown *db/db* mice; however, this did not reach statistical significance. Despite only 30%–35% knockdown of Snhg12 detected in RNA extracted from gastrocnemius muscle after 2 weeks of biweekly injections, immunofluorescence from gastrocnemius muscle ipsilateral to FAL showed reduced vessel diameter (by 38%) and vessel number (by 83%) in comparison with controls. Moreover, the phenotype also included decreased total CD31 fluorescence (by 76%) in Snhg12-knockdown *db/db* mice, implying a more severe angiogenic phenotype in this diabetic model compared with C57BL/6 mice (Figure 6D and Figure 3E). Despite increased baseline levels of inflammation in diabetic models of obesity, there was no difference in tissue accumulation of leukocytes as measured by CD45 and no changes in M1-like or M2-like macrophage polarization as measured by CCR7 or CD206 expression by immunofluorescence (Supplemental Figure 9D).

### Transcriptomic profiling reveals novel insights into the mechanism of SNHG12’s effects on angiogenesis in vitro.

**Figure 7:**
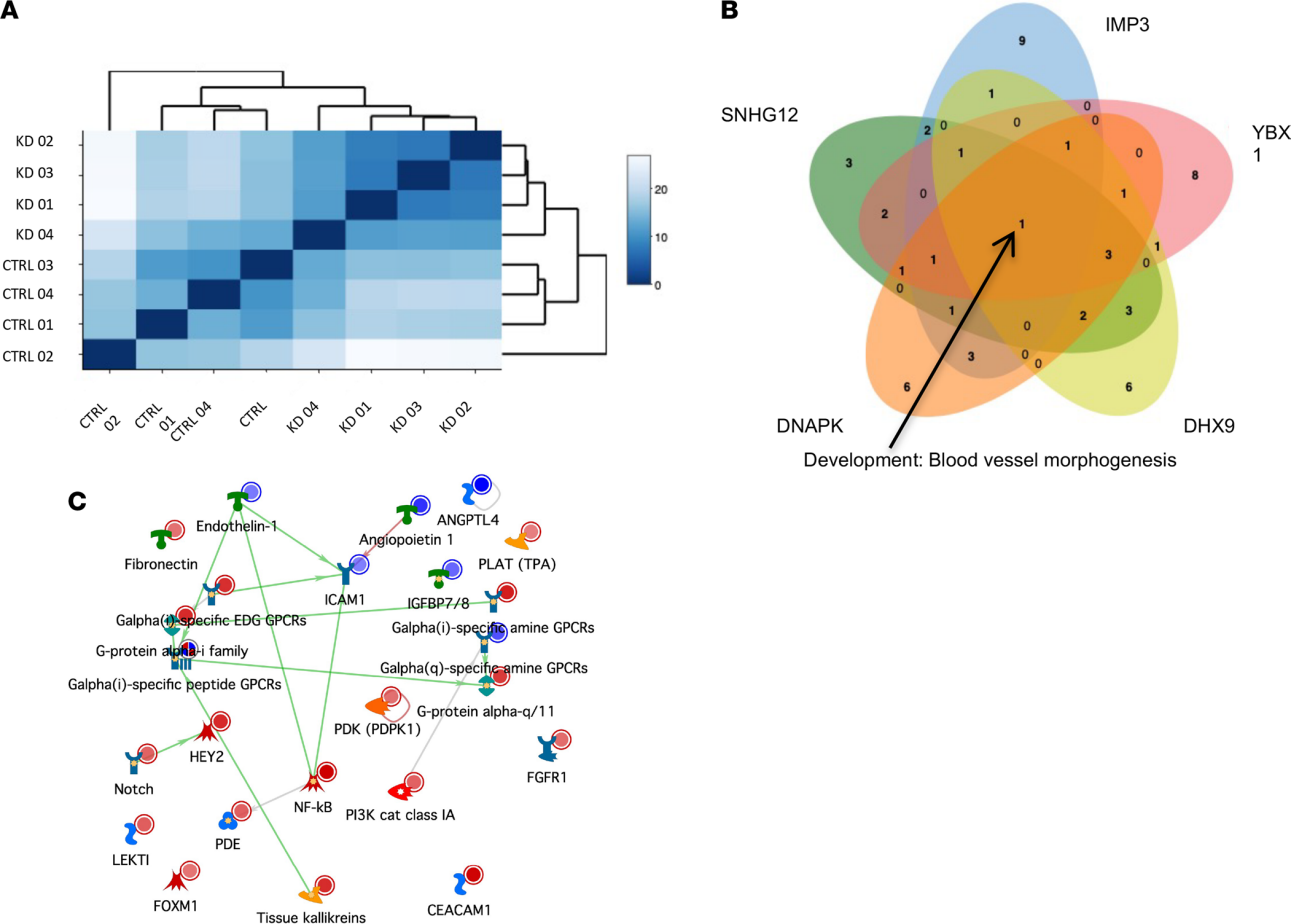
RNA-Seq analysis of SNHG12 and its interacting proteins in vitro. (**A**) Hierarchical clustering plot from genome-wide RNA-Seq transcriptomic profiling of HUVECs transfected with control or SNHG12 gapmeR (*n* = 4 per group). (**B**) MetaCore process network analysis of HUVECs with knockdown of SNHG12, IMP3, YBX1, DHX9, or DNAPK was performed. The top 20 hits were overlaid, and only blood vessel morphogenesis was found to be a common network among SNHG12 and its interacting proteins. (**C**) Gene network visualization of the “development: blood vessel morphogenesis” pathway in HUVECs with knockdown of SNHG12 shows decreased expression of angiogenic factors angiopoietin-1, ANGPTL4, endothelin-1, ICAM1, and IGFBP7/8 (blue targets indicate downregulation and red targets indicate upregulation).

In order to glean insight into the mechanism by which SNHG12 abrogates angiogenesis in vitro and in vivo, we first performed transcriptomic profiling in HUVECs stimulated with VEGF in a manner similar to conditions used for angiogenic sprouting assays. Evaluation of published RNA-Seq data using Ingenuity Pathway Analysis (IPA) from earlier work on SNHG12 in our laboratory showed that VEGF is likely a critical node in mediating the effects of SNHG12 on atherosclerosis (21). Knockdown of SNHG12 in HUVECs using gapmeRs revealed high correlations among samples from the same treatment group (<0.999) in hierarchical clustering plots (Figure 7A). In order to understand how knockdown of SNHG12 and its interacting proteins functioned to reduced angiogenesis, we performed RNA-Seq in HUVECs with knockdown of SNHG12-interacting proteins as well. Using MetaCore (Clarivate Analytics), we overlaid the top 20 process networks to identify commonly dysregulated networks. The only overlapping dysregulated process network was “blood vessel morphogenesis” (Figure 7B). Gene network visualization of this pathway in HUVECs with knockdown of SNHG12 suggested that this was driven by angiopoietin-1, ANGPTL4, IGFBP7/8, ICAM1, and endothelin-1 (Figure 7C). A volcano plot labeled for the 20 most significantly differentially expressed genes identified downregulation of cyclin 3 (CLCN3) and increased DRAXIN, a potent Wnt antagonist and antiangiogenic protein (Supplemental Figure 10) (29). Given that pathway analysis and volcano plots derived from SNHG12 knockdown data sets demonstrated dysregulation of several angiogenic (e.g., ephrin, Wnt, angiopoietin, endothelin-1) and non-angiogenic pathway components (e.g., GPCRs, PDK1) (Figure 7C and Supplemental Figure 10), we hypothesized that suppression of angiogenesis may be driven by a more fundamental process such as cell cycle regulation and cell growth and proliferation.

**Figure 8:**
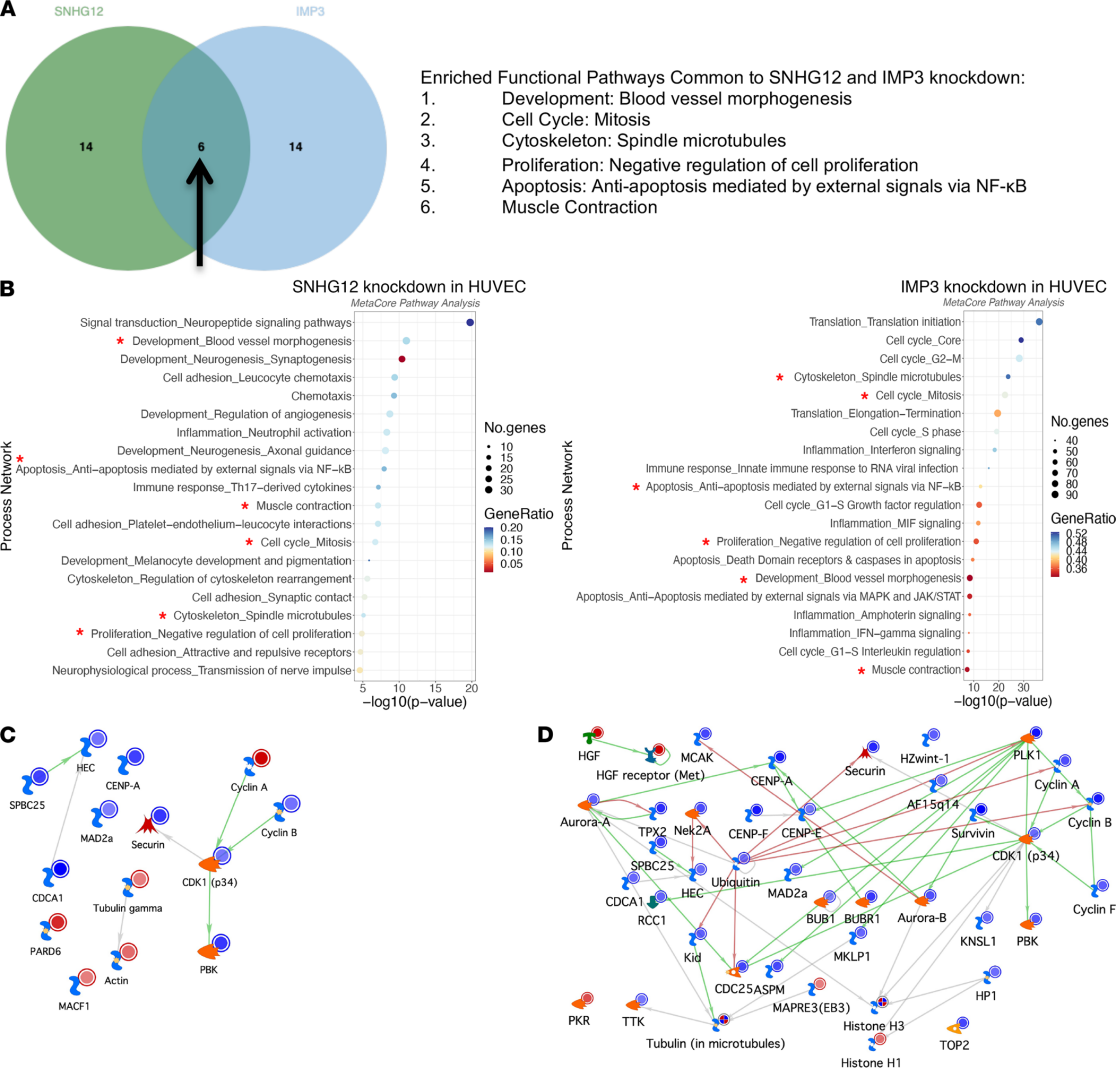
IMP3 is a novel SNHG12-interacting protein. (**A**) Venn diagram of overlapping top 20 MetaCore process networks shows commonality in cell cycle regulation, an overlap in RNA-Seq data set process network analysis that is distinct from other SNHG12-interacting proteins. (**B**) Gene set enrichment analysis using MetaCore process networks of SNHG12 versus control gapmeR–mediated knockdown in HUVECs (left) and IMP3 versus control siRNA–mediated knockdown (right). Red stars indicate overlapping process networks as noted in **A**. Process networks are graphed in dot plot format arranged in descending level of significance by –log_10_(*P* value) with dot size representing the number of genes from a process network and color representing the gene ratio (percentage) of significantly differentially expressed genes from that network. Red stars indicate overlapping process networks as shown in **A**. (**C**) Gene network visualization of “cell cycle: mitosis” pathway shows a central node of CDK1 (p34) in SNHG12 knockdown in HUVECs. (**D**) Gene network visualization of “cell cycle: mitosis” pathway shows a central node of CDK1 (p34) in IMP3 knockdown in HUVECs, also with decreased expression of SPBC25, HEC, MAD2a, CENP-A, securin, cyclin B, and PBK, as was noted in HUVECs with knockdown of SNHG12.

Overlapping process networks between SNHG12 and its interactors revealed a distinct effect on cell cycle networks shared by SNHG12 and IMP3 (Figure 8A). The 2 pathways that were unique in overlap to SNHG12 with IMP3 were “cell cycle: mitosis” and “cytoskeleton: spindle microtubules” (Figure 7B and Figure 8, A and B). Contrarily, the unique pathways between SNHG12 and YBX1 comprised “neurogenesis_axonal guidance” and “attractive and repulsive receptors,” and the distinct pathways shared with DHX9 included “Th17-derived cytokines,” “melanocyte development and pigmentation,” and “regulation of cytoskeleton rearrangement” (Supplemental Figure 11, A and B). Gene set enrichment analysis (GSEA) using process networks from MetaCore Pathway Analysis showed that the common pathways dysregulated between SNHG12 and IMP3 were all highly significant (Figure 8B). These enrichments showed that IMP3 had the most significant differential effects on cell cycle pathways (Figure 8B). Visualization of gene networks of the “cell cycle: mitosis” process network showed that CKD1 (p34) was a central hub that had significantly reduced expression under conditions of SNHG12 or IMP3 knockdown (Figure 8, C and D, respectively). Overlapping cell cycle proteins that were decreased in their expression include cyclin B, securin, PBK, CDCA1, SPBC5, HEC, CENP-A, and MAD2a. The top 20 process networks identified by MetaCore Pathway Analysis of HUVECs with knockdown of YBX1, DHX9, or DNAPK revealed that blood vessel morphogenesis was highly significant among HUVECs with knockdown of each SNHG12 interactor (Supplemental Figure 11), but relatively more significant compared with other networks for YBX1 and DHX9 knockdown. Together, these data suggest a distinct effect on cell cycle networks shared by SNHG12 and its interacting partner IMP3.

### Transcriptomic profiling from ischemic diabetic mouse gastrocnemius muscle demonstrates an antiangiogenic effect of Snhg12 knockdown.

**Figure 9:**
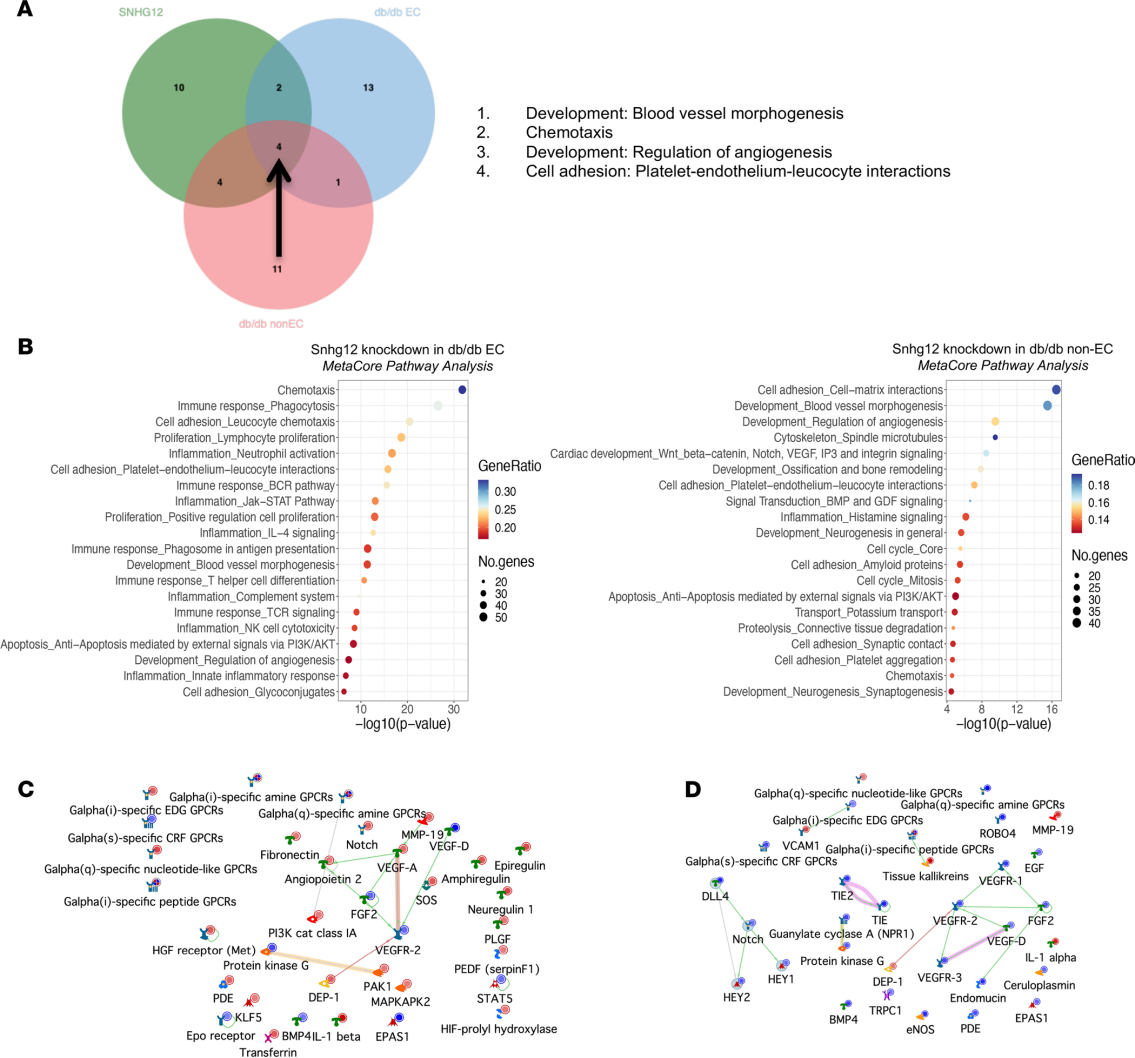
RNA-Seq analysis of Snhg12 knockdown in *db/db* gastrocnemius. (**A**) Venn diagram of overlapping process networks between RNA-Seq data sets of SNHG12 knockdown in HUVECs and Snhg12 knockdown in *db/db* mouse gastrocnemius EC and non-EC fractions yields blood vessel morphogenesis, chemotaxis, regulation of angiogenesis, and platelet-endothelium-leukocyte interaction process networks. (**B**) Gene set enrichment analysis using MetaCore process networks in *db/db* EC (left) and *db/db* non-EC (right) gastrocnemius cell fractions. (**C**) Gene network visualization of “development: blood vessel morphogenesis” in *db/db* gastrocnemius ECs shows significant downregulation of VEGF and FGF pathway nodes and significant upregulation of the antiangiogenic MMP19. (**D**) Gene network visualization of “development: blood vessel morphogenesis” in *db/db* gastrocnemius non-ECs shows significant downregulation of VEGF, FGF, and EGF signaling pathways in addition to VEGF receptor Tie2, Tie1, eNOS, Notch, and BMP pathways and significant upregulation of the antiangiogenic MMP19.

In an effort to delineate specific antiangiogenic pathways dysregulated in ischemic diabetic mouse muscle, we performed transcriptomic profiling of gastrocnemius ECs and non-ECs from the *db/db* mice that underwent FAL as shown in Figure 6. Overlap analysis of process networks between HUVECs with knockdown of Snhg12 and EC and non-EC muscle fractions with knockdown of Snhg12 using MetaCore showed that blood vessel morphogenesis, regulation of angiogenesis, chemotaxis, and platelet-endothelium-leukocyte interactions constituted the shared significantly differentially expressed networks (Figure 9A). GSEA showed that Snhg12 knockdown in the *db/db* gastrocnemius EC population identified a higher relative importance of process networks including cellular proliferation and inflammatory signaling networks (Figure 9B). However, GSEA of the non-EC gastrocnemius fraction noted greater relative emphasis on the pathways of blood vessel morphogenesis and regulation of angiogenesis (Figure 9B). Interestingly, this was despite a similar percentage knockdown in both populations and lower relative Snhg12 expression in this non-EC population (Figure 6C). Gene network visualization of blood vessel morphogenesis in the *db/db* EC population using MetaCore showed downregulation of Vegf, Fgf, and Bmp genes and drastic upregulation of Mmp19, a potent angiogenesis antagonist (Figure 9C). In parallel processing of transcriptomic data using IPA, we recognized that there was also a heavy gene set weighting toward Notch and Wnt signaling pathway genes among the most significant gene sets, such as “breast cancer regulation by stathmin1,” “CREB signaling in neurons,” “hepatic fibrosis signaling pathway,” “tumor microenvironment pathway,” “basal cell carcinoma signaling,” and “hepatic stellate cell activation” (Supplemental Figure 12A). Volcano plots showed that Tmem230, which modulates Delta/Notch pathway signaling and is involved in the regulation of EC proliferation (30), and Polg2, a DNA polymerase subunit, were among the most significantly downregulated genes (Supplemental Figure 12B). Interestingly, the epithelial-mesenchymal transition gene Glipr2 was induced by Snhg12 knockdown (Supplemental Figure 12B) (31). There was also increased expression of antiangiogenic Dpysl3, which has been observed to inhibit angiogenesis in hepatocellular carcinoma cell lines (32); and Lif, long known to inhibit Vegf and Fgf stimulation of angiogenesis, was overexpressed (33). Furthermore, the antiproliferative transcription factor Rfx1 (34) and tumor suppressor Rassf2 (also found to be regulated by a microRNA studied in our laboratory, miR-615-5p) (35) demonstrated increased expression (Supplemental Figure 12B). Querying Notch and Wnt pathways and various angiogenic transcription factors, canonical Wnt signaling genes emerged with reduced expression upon Snhg12 knockdown (Supplemental Figure 12C). Despite no clear expression differences in upstream Notch signaling, expression of Hey2, a transcriptional target of this pathway, decreased, suggesting that Snhg12 modulates selective parts of the Notch pathway (Supplemental Figure 12C). While IPA analysis also identified decreased expression of Bmp, endothelin, and Vegf/Vegfr, there was increased antiangiogenic Mmp19 expression (Figure 9D). Furthermore, IPA highlighted the downregulation of several transcription factors — Gli1, Lhx2, and Gata3 — known to be involved in angiogenesis or cellular proliferation (Supplemental Figure 12C) (36, 37).

While transcriptional network assessment of the *db/db* EC population suggested decreased Wnt and a trend toward decreased Notch signaling, gene network analysis of the *db/db* non-EC population showed a clear effect on the expression of Notch pathway components using IPA analysis and identified decreased apelin pathway expression (Supplemental Figure 13A). A volcano plot of *db/db* non-EC transcriptional data showed that Dll4 (38, 39), Rapgef5 (a Dll4 target) (40), Pecam1 (CD31), Tie1, Apln5 (41), laminin C3 (42), Cntn1 (43), and Dipk2b (44) were among the 20 most significantly differentially expressed genes, implicating a variety of aspects of angiogenesis that are decreased by SNHG12 knockdown. Overall, the non-EC cell population from *db/db* mice more strongly demonstrated downregulation of the Wnt and Notch pathways and associated transcription factors, in addition to decreased Vegf/Vegfr (Supplemental Figure 13C). Taken together, these findings in the *db/db* EC and non-EC cell populations indicate that Snhg12 acts to potently decrease angiogenesis through a variety of mechanisms, particularly involving cellular proliferation and Wnt, Notch, and Vegf signaling pathways.

## Discussion

Impaired angiogenesis is a hallmark of PAD. Various lncRNAs have been identified as being dysregulated in ECs in response to hypoxia, high-glucose conditions, nonlaminar flow, and EC differentiation (12–17). LncRNAs also play important roles in angiogenesis by mechanisms that include repressing expression of VEGF-responsive microRNAs, regulating cyclins and cell cycle progression, affecting chromatin remodeling, and modulating proangiogenic mRNA levels (13, 14). Our group recently identified *SNHG12* as an important regulator of vascular senescence, and decreased *SNHG12* expression leads to increased p21 and p16 expression, markers of the senescent phenotype, as well as markers of increased DNA damage, such as γH2AX (21). However, its role in regulating angiogenesis remained unknown. This study provides evidence, for the first time to our knowledge, that SNHG12 deficiency impairs the angiogenic response to ischemia.

Vascular senescence occurs with aging, has been shown to be deleterious in atherosclerosis and angiogenesis progression, and is driven by signaling driven by the senescence-associated secretory proteome (45, 46). This often involves telomere shortening, accrual of DNA damage, and progressive mitochondrial dysfunction, which leads to increased reactive oxygen species and decreased nitric oxide production and increased p53, p16, and p21 expression and activation, ultimately culminating in impaired endothelium vasodilation, increased thrombogenesis, accelerated atherosclerosis, and reduced angiogenesis (47). Dysregulation of these processes is linked to human progeria syndromes in which patients often experience accelerated peripheral arterial disease and die from myocardial infarction in part due to accelerated atherosclerosis (48). Little is known about the roles of these pathways in angiogenesis and the ischemic response to hind-limb ischemia. Recently, it was shown that *p53^–/–^* mice had improved blood flow recovery after FAL with increased capillary density in lower extremity muscle, with increased HIF1a and VEGF expression in ischemic tissue, effects that were recapitulated in vitro (49). While no hind-limb ischemia studies have been performed on *p21^–/–^* or *p16^–/–^* mouse models, knockdown of the lncRNA *H19* has been shown to affect senescence with increased p16 and p21 expression and decreased cellular proliferation, leading to the accumulation of cells in G_0_/G_1_ phase (19).

Aging also impacts skeletal muscle, contributing to a progressive decline in muscle size, myofiber area, and muscle strength and function. Several clinical studies in PAD patients have demonstrated that muscle function or exercise capacity is a strong predictor of morbidity/mortality (50, 51). Notably, knockdown of SNHG12 was found to increase the proportion of small myofibers and an expansion of the non-myofiber area, two features that hinder muscle force development and are indicative of a significant ischemic myopathy. It is unknown at this time whether the exacerbated ischemic myopathy with SNHG12 gapmeR treatment stems from direct effects in the myofibers, is linked to the impaired limb perfusion and angiogenesis caused by knockdown of SNHG12, or a combination of both. Nevertheless, these features of ischemic myopathy and impaired limb hemodynamics are consistent with more severe symptomatic PAD/CLI.

Our study builds on a growing body of literature implicating lncRNAs as pivotal regulators of angiogenesis and tissue ischemia responses. Compared with Snhg12, various lncRNAs have been found to be significantly upregulated under hypoxic conditions of hind-limb ischemia (including Meg3, Malat-1, and H19) (52). SNHG12 demonstrates what appears to be a biphasic response, differing from the paradigm of Malat-1, which was identified to be upregulated under conditions of hind-limb ischemia. In fact, early after FAL, Snhg12 expression decreases, but at later time points, Snhg12 expression significantly increases in C57BL/6 mice. Indeed, in an alternative model of hind-limb ischemia using BALB/c mice, Snhg12 expression significantly increases within 1 week of hind-limb ischemia and begins to normalize at later time points. It is unknown whether similar kinetics are observed in human cohorts with CLI. It is interesting to note the variability of Snhg12 expression kinetics between C57BL/6, BALB/c, and *db/db* mice, a finding that may suggest that Snhg12 is more important in the angiogenesis response to hind-limb ischemia in BALB/c and *db/db* mice, where no significant decreases in Snhg12 expression were observed after FAL. *MALAT-1* displays a regulation of cell cycle genes similar to that seen with SNHG12: SNHG12 significantly attenuated expression of Cdks 1, 2, and 17 and cyclins A2, B1, B2, C, E2, H, and I; increased p21; and decreased elongation factors E2F1, E2F6, and E2F8 without significant transcriptional effects on Rb1 or p53, which was similar to the effects of MALAT1 (12). While MALAT-1 demonstrated an antiproliferative but promigratory state, SNHG12 abrogation resulted in decreased proliferation and cellular migration. Interestingly, Snhg12 was also identified as being upregulated in a model of cerebral ischemic injury under glucose deprivation and hypoxic conditions, and knockdown in brain ECs was found to increase the expression of proinflammatory markers, which was nullified by overexpression. By overexpressing Snhg12 after middle cerebral artery occlusion in mice, Zhang and colleagues were able to show decreased infarct volumes (53, 54). It is possible that the biphasic response of Snhg12 could play a role in the proinflammatory response soon after FAL, but its more predominant role in later phases is with respect to its effects on angiogenesis and cell cycle regulation.

Interestingly, Snhg12 knockdown in the *db/db* model relative to C57BL/6 resulted in an exaggerated reduction in arterial diameter and number of arteries per high-power field. Unexpectedly, significant reduction in CD31 staining was observed in *db/db* but not in C57BL/6 mice. It is tempting to speculate that decreased arterial staining by immunofluorescence in C57BL/6 mice may be due to a dysfunctional vasodilator response to hypoxia. It is also possible that *db/db* mice have a more significant reduction in vessel diameter, number, and CD31 staining due to more profound impairments in tissue repair and wound healing mechanisms compared with C57BL/6 mice at baseline, a finding commonly observed in CLI patients with diabetes versus those without.

When SNHG12 knockdown was compared with knockdown of verified SNHG12 interactors in an effort to assign responsibility for this phenotype to a protein interaction, transcriptional profiles of SNHG12 knockdown were most similar to those of IMP3 knockdown relative to other SNHG12 interactors in HUVECs. Cell cycle control was notably overrepresented among top gene set enrichment terms upon IMP3 knockdown. Taken together, these data suggest that SNHG12’s interaction with IMP3 controls the proliferative phenotype caused by SNHG12 modulation. Unfortunately, little is known about how IMP3 controls proliferation as an mRNA-binding protein in the nucleus, despite its having 2 homologs, IGF2BPs 1 and 2 (also known as IMP1 and IMP2). IMP3, which is primarily a cytosolic oncofetal protein, is thought to be loaded onto mRNA targets in the nucleus, and has been shown to be important for invadopodia formation, cell adhesion, and cell proliferation (55, 56). Interestingly, IMP1 has been shown to be a target of the β-catenin signaling transcription factor Tcf, which subsequently binds to and stabilizes the Gli1 Hedgehog pathway transcription factor, linking it to the Wnt signaling pathway (36, 57). Gli1 has also been linked to the expression of VEGF-C and bFGF (58). While much work has been performed on SNHG12 and its dysregulation in multiple cancer cell lines and tumor samples, little is known about its function in the vasculature.

DNAPK has previously been shown to phosphorylate RNA helicase A (DHX9, previously known as NDH II) and can function to regulate some aspects of RNA metabolism (59). DNAPK binds to DHX9 in cells, forming a complex with hnRNP A1 in the nucleus (60). Given the similar transcriptional profiles between DNAPK, DHX9, and Ybx1, it is tempting to speculate that Ybx1 might associate with DNAPK and DHX9 in order to form a complex given that all three cluster with one another in both PCA and hierarchical clustering. Unfortunately, little is known regarding the function and mechanisms of IGF2BP3 and Ybx1 in RNA biology, let alone angiogenesis, making conclusions regarding the precise mechanism by which SNHG12 mediates angiogenesis limited (61, 62). While we identified 3 binding partners in addition to DNAPK, it is plausible that SNHG12 has a different set of binding partners that are influenced by cell cycle state, growth conditions, or cell lineage and time in development. It is notable that the expression of SNHG12 interactors (IMP3, YBX1, DHX9, DNAPK) is significantly decreased in the human CLI cohort, whereas SNHG12 expression is increased. A possible explanation for this is that the regulation of IMP3, YBX1, DHX9, and DNAPK is likely driven by a different pathway than the regulation of SNHG12.

The effect of Snhg12 on angiogenesis in *db/db* mice more clearly demonstrates that multiple bona fide angiogenic pathways are downregulated at various control nodes. Angiogenesis canonically depends on intact Notch, Wnt, BMP/SMAD, angiopoietin, and VEGF signaling and has more recently been shown to involve additional pathways such as apelin signaling. For example, Dll4 is known to control sprouting angiogenesis modulated by the Notch pathway and is a key factor important for tip and stalk cell communication (63). The profound transcriptional downregulation of several angiogenic pathways suggests that Snhg12 regulates a more fundamental process that leads to decreased signaling through each pathway, possibly through regulation of cell cycle progression. Importantly, inflammation, often observed to be involved in the recruitment of monocytes and macrophages to aid in the angiogenic response, was not noted to be increased histologically. However, our data are not able to exclude the possibility that macrophage-EC communication may be altered in Snhg12-expressing versus Snhg12-knockdown cells.

A limitation of this study is due to the use of gapmeRs, which must be repeatedly administered in order to maintain knockdown of Snhg12 throughout physiologic experimentation and display significant variability between animals. This is in contrast to genetic strategies like CRISPR/Cas9 editing for genetically engineering mouse models. To mitigate the possibility of a phenotype being due to intravenous injection and effect on gapmeR delivery on hepatocytes (which clear gapmeRs) and PBMCs, we used intramuscular injection, which allowed optimized delivery to the ischemic vasculature. To our knowledge, this is the first time an lncRNA has been delivered intramuscularly to the ischemic leg (8). It is possible that this could be contributory to why an inflammatory phenotype with monocyte and macrophage infiltration was not seen histologically. Furthermore, it is probable that the degree of knockdown efficiency is important for ischemic phenotype severity. An Snhg12 knockout in a tissue-specific manner would allow specific determination of EC-restricted effects and allow an understanding of the interplay with the non-EC compartment. Given the drastic effects of Snhg12 on angiogenesis, it is tempting to speculate that systemic or non-inducible knockout might be embryonic-lethal as has been the case for multiple mouse models exploring the essential nature of lncRNAs (64). However, as is the case with many lncRNAs, the efficiency of knockdown that induces a significant phenotype can range from 50% to 75% and is thought to depend on relative lncRNA cellular abundance and whether it acts in *cis* or in *trans* (13). Given the kinetics of Snhg12 expression in the context of acute limb ischemia caused by FAL, it is possible that a better mouse model for human CLI patients is a chronic limb ischemia model using ameroid constrictors; however, this approach is significantly more complicated and increases experimental variability (65). Future studies will be of interest to assess the specific upstream drivers of SNHG12 regulation at the promoter and the transcript levels and whether this is a regulatory effect on lncRNA stability.

In conclusion, we have demonstrated here that Snhg12 is dysregulated after hind-limb ischemia and plays an important role in angiogenesis and the ischemic angiogenic response to hind-limb ischemia. The identification of IMP3 as a previously unrecognized SNHG12-binding protein provides clues to the mechanism by which SNHG12 leads to decreased expression of cell cycle regulatory genes. Knockdown of Snhg12 in vivo using intramuscular delivery of gapmeRs in *db/db* mice shows that there is a significant abrogation of the expression of multiple angiogenic pathway components from the Wnt, Notch, and VEGF signaling pathways. Further investigations using cell-specific genetically engineered mouse models will allow better dissection of the importance of these pathways in the mechanism by which SNHG12 is involved in angiogenesis.

## Methods

Additional detailed methods can be found in Supplemental Methods.

### Angiogenesis and cell proliferation assays.

BrdU assays were performed by transfection of HUVECs with gapmeR (25 nM) or transduction of HUVECs with lentivirus. After 24–36 hours, cells were seeded at 5000 cells per well in 96-well plates, and 24 hours later, they were labeled with BrdU reagent for 6–8 hours, fixed, and quantitated using the Cell Proliferation ELISA BrdU Colorimetric Kit according to the manufacturer’s instructions (Sigma-Aldrich, 11647229001).

Scratch assays were performed by transfecting 10^5^ HUVECs with gapmeR (25 nM) or siRNAs (10 nM) or transducing HUVECs with lentivirus and seeding them at 25,000 cells per well in μ-dish glass plates (Ibidi Culture-Insert 3-well μ-dish, 501149017). When cells were confluent, well dividers were removed, and the resultant “scratch” was imaged at time 0 and various time points up to 24 hours, at which time “wounds” were closed.

For spheroid sprouting assays, 10^5^ HUVECs were transfected with gapmeRs or siRNAs or transduced with lentivirus in 6-well plates. After 24 hours of transfection or transduction, cells were harvested in EGM2 media (Lonza), without growth factors, containing 0.2% methylcellulose (Sigma-Aldrich), resuspended at 10^3^ cells per 25 μL droplet, plated on the lid of a 25 cm plate, stored upside down (hanging droplet method), and placed at 37°C in a cell culture incubator overnight. The following day, hanging droplet HUVEC spheroids were collected by pipetting of 5–10 mL of PBS with 10% FBS onto each lid, swirled gently, and transferred to 50 mL conical tubes and centrifuged at 300*g* for 5 minutes at room temperature without brake. The supernatant was carefully aspirated, and the pellet was overlaid and very gently pipetted up and down with a premixed methylcellulose mixture containing 1% methylcellulose in 40% FBS, 2.34 mg/mL NaHCO_3_, 30% type I rat collagen (Cultrex, 3447-020-01), and 10 mM NaOH. Five hundred microliters of this spheroid-methylcellulose mixture was aliquoted to individual wells of a 24-well plate and allowed to polymerize for 5 minutes at 37°C. Embedded spheroids were overlaid with 500 μL EGM2 media containing growth factors and 100 ng/μL VEGF (for a final concentration of 50 ng/μL VEGF per well). Cells were placed at 37°C overnight, and imaging was performed on a Nikon inverted light microscope using ×4 or ×10 objectives.

Transwell migration assays were performed by seeding of 37,500 HUVECs in 300 μL EBM2 media that had been transfected with gapmeR or transduced with lentivirus 48 hours earlier into QCM Chemotaxis Cell Migration Assay, 24-well, 8 μm pore size, following the manufacturer’s protocol (EMD Millipore).

### Femoral artery ligation.

Mice that were pretreated with 2 consecutive days of tail vein or intramuscular gastrocnemius gapmeR injection were subjected to femoral artery ligation (FAL) as described by Wara et al. (66, 67). Briefly, mice were injected i.p. with 150 μL of a mixture of 20% ketamine/5% xylazine in 0.9% saline. Once anesthetized, the right medial thigh to the suprapubic area was treated with a commercial emollient to remove fur and sterilized with povidone-iodine. Skin and fascia were dissected away to the femoral bed. The femoral artery and adjacent muscle were visualized and proximally and distally ligated with 7-0 Prolene sutures. The arterial bed in between sutures was cauterized. Abrogation of blood flow in comparison with the contralateral limb (<10%) was confirmed using a laser Doppler imager (Moor Instruments, UK). Once confirmed, mice were then sutured closed at the level of the fascia and, subsequently, the skin. Sham-treated mice were treated the same way except that once the femoral artery was visualized, the incision was closed without ligation of the femoral artery or cauterization. Percentage blood flow recovery was calculated as the ratio of ischemic paw to contralateral paw Doppler count profiles, and normalized blood flow recovery was calculated by comparison of this ratio versus day 0 postoperative percentage blood flow. In some studies, animals were injected with 200 μg *Lycopersicon esculentum* FITC-lectin (1 mg/mL; Sigma-Aldrich) 30 minutes before sacrifice via tail vein injection in order to label perfused capillary beds.

### Tissue oxygen measurements.

Mice anesthetized with 20% ketamine/5% xylazine were placed on a 37°C heating pad, and after imaging of blood flow by laser Doppler imaging, 23-gauge needles were inserted at approximately the mid-gastrocnemius (1 per gastrocnemius) at the widest portion of the muscles. Through the needles, the OxyLite Pro (Oxford Optronics Ltd., UK) Oxygen/Temperature Bare-Fiber Sensor (Scintica) was introduced into bilateral gastrocnemius muscles (allowing about 4–5 mm of the probe to be sitting in gastrocnemius muscle). The OxyLite Pro system measures partial pressure of oxygen (pO_2_) by luminescence quenching. Once inserted at 4–5 mm depth from the skin, the sensor was allowed about 10 minutes to equilibrate, and 30 seconds of pO_2_ measurements were averaged for each limb (ischemic and contralateral) at one location so as not to create additional tracts in the muscle that might affect histology or intramuscular injections.

### Endothelial cell isolation.

Magnetic Dynabeads (sheep anti-rat IgG, Invitrogen, 00412289) were prewashed 5 times in PBS with 0.1% BSA and then incubated with rat anti–mouse CD31 antibody (BD Biosciences, 557355) at a ratio of 5:1 Dynabeads/antibody overnight at 4°C in a tube rotator. A 15 μL mixture was prepared per tissue requiring EC isolation. These were then washed again in PBS with 0.1% BSA 5 times and set aside at 4°C for subsequent use. Gastrocnemius muscle was dissected away from soleus muscle and washed in PBS. Subsequently, the tissue was placed in 1 mL Digestion Buffer (1 mg/mL collagenase type II [Worthington Biochemical, LS004177], 1 mg/mL dispase II [Roche, 04942078001] in DMEM/F-12 medium [Life Technologies, 11320033]) and minced with scissors for 1 minute. Minced tissue was transferred to a 15 mL Falcon tube containing 9 mL additional Digestion Buffer and incubated at 37°C for 40 minutes with vigorous shaking using a test tube rocker every 10 minutes. The slurry was then passed through a 70 μm cell strainer (Corning Falcon/Westnet, 352350), and 10 mL of Wash Buffer 2 (DMEM/F-12 with 10% FBS) was added. The slurry was then centrifuged at 500*g* for 10 minutes at 4°C.

The supernatant was aspirated and discarded. The residual pellet was manually disrupted, and 1 mL RBC Lysis Buffer was added (eBioscience, 00-4300-54) for 5 minutes at room temperature with occasional gentle shaking. The RBC Lysis reaction was neutralized with 10 mL Wash Buffer 2 and then passed through a 40 μm cell strainer (Corning Falcon/Westnet, 352340). The eluate was centrifuged at 500*g* for 10 minutes at 4°C, and the resulting pellet was suspended in 1 mL of Wash Buffer 1 (PBS pH 7.2, 0.1% BSA, 2 mM EDTA, 0.5% FBS). The solubilized pellet was added to a 15 μL prewashed and conjugated Dynabead/CD31 antibody mixture and allowed to tumble at 4°C for 20 minutes. The slurry of lysate and Dynabead/antibody mixture was bound on a Dynamag-2 Magnet (Invitrogen) for 1 minute, and the supernatant was collected as a non-EC fraction. This supernatant was pelleted, and the pellet was saved at –80°C in 0.5 mL Trizol (Invitrogen) for downstream analysis (i.e., RNA isolation for quantitative RT-PCR). The beads containing bound ECs were then washed on the Dynamag-2 Magnet 5 times, and the resultant pellet was eluted in 0.5 mL Trizol and saved at –80°C for downstream analysis.

### RNA-Seq analysis.

RNA-Seq analysis was performed after ribodepletion and standard library construction using Illumina HiSeq2500 V4 2×100 PE (Genewiz). All samples were processed using an RNA-Seq pipeline implemented in the bcbio-nextgen project (https://bcbio-nextgen.readthedocs.io/en/latest). Raw reads were examined for quality issues using FastQC (http://www.bioinformatics.babraham.ac.uk/projects/fastqc/) to ensure that library generation and sequencing were suitable for further analysis. Trimmed reads were aligned to UCSC build mm10 of the mouse genome and augmented with transcript information from Ensembl releases 86 (*H*. *sapiens*) and 79 (*M*. *musculus*) using STAR (59). Alignments were checked for evenness of coverage, rRNA content, genomic alignment context, and other quality checks using a combination of FastQC and Qualimap (60). Counts of reads aligning to known genes were generated by featureCounts (61). Differential expression at the gene level was called with DESeq2 (62). Total gene hit counts and counts-per-million values were calculated for each gene, and downstream differential expression analysis between specified groups was performed using DESeq2 and an adapted DESeq2 algorithm that excludes overlapping reads. Genes with adjusted FDR less than 0.05 and log_2_ fold change greater than 1.5 were called as differentially expressed genes for each comparison. The mean quality score of all samples was 35.91 with a range of 28 × 10^6^ to 63 × 10^6^ reads per sample. All samples had greater than 96% of mapped fragments over total fragments. Coverage was visualized using Integrative Genomics Viewer (version 2.3.68). Ingenuity Pathway Analysis (IPA; QIAGEN) and MetaCore (version 20.2, Clarivate Analytics) were used for functional GSEA. RNA-Seq data were deposited in the NCBI’s Gene Expression Omnibus database (accession GSE188875, GSE188876, and GSE188877).

### Statistics.

Data are shown as the mean ± SEM. Statistical differences were calculated using unpaired 2-tailed Student’s *t* test or 1-way ANOVA with Bonferroni correction for multiple comparisons. A *P* value of less than 0.05 was considered significant. Outliers were removed using Dixon’s *Q* test. For illustration of differentially expressed genes, Microsoft Excel and GraphPad Prism were used.

### Study approval.

All protocols concerning animal use were approved by the Institutional Animal Care and Use Committee at Brigham and Women’s Hospital and Harvard Medical School and conducted in accordance with the NIH *Guide for the Care and Use of Laboratory Animals* (National Academies Press, 2011). Studies were performed in male *db/db* and BALB/c mice (The Jackson Laboratory) and C57BL/6 mice (Charles River).

## Author contributions

DAG, TER, and MWF designed research studies and wrote the manuscript. DAG, HSC, RZ, MGM, DPC, ZS, SH, TER, and AKMKW conducted experiments, acquired data, and analyzed data. SH provided reagents.

## Supplementary Material

Supplemental data

## Version 1 11/18/2021

In-Press Preview

## Version 2 01/11/2022

Electronic publication
