# Supplementary material for: Deficiency of lncRNA SNHG12 impairs ischemic limb neovascularization by altering an endothelial cell cycle pathway
Source: JCI Insight. 2022 Jan 11;7(1):e150761. doi: 10.1172/jci.insight.150761 (PMC8765056; doi:10.1172/jci.insight.150761)
Supplement: Supplemental data [file jciinsight-7-150761-s120.pdf]

Deficiency of lncRNA SNHG12 impairs ischemic limb neovascularization by altering an endothelial cell cycle pathway

David A. Gross[1], **Henry S. Cheng**[1], **Rulin Zhuang**[1, 2], **Michael G. McCoy**[1], **Daniel Pérez-Cremades**[1,3], **Zachary Salyers**[4], **A.K.M. Khyrul Wara**[1], **Stefan Haemmig**[1], **Terence E. Ryan**[4], **Mark W. Feinberg**[1]*

[1] Department of Medicine, Cardiovascular Division, Brigham and Women's Hospital, Harvard Medical School, Boston, MA, USA

[2] Department of Cardiovascular Surgery, Shanghai East Hospital, Tongji University School of Medicine, Shanghai, 200120, China

[3] Department of Physiology, University of Valencia and INCLIVA Biomedical Research Institute, Valencia, Spain

[4] Department of Applied Physiology & Kinesiology, University of Florida, Gainesville, FL, USA

* **Correspondence to** Mark W. Feinberg, MD, Department of Medicine, Cardiovascular Division, Brigham and Women's Hospital, Harvard Medical School, Louis Pasteur Avenue 77, 02115, Boston, Massachusetts, USA, E-mail: mfeinberg@bwh.harvard.edu, Tel: (617) 525-4381

Supplemental Methods

Cell Culture and Transfection

Human umbilical vein endothelial cells (HUVECs; Lonza) were cultured in endothelial cell growth medium EGM-2 (Lonza). Cells that were utilized for experiments were passaged no more than six times. Peripheral blood mononuclear cells (PBMCs) were isolated by overlaying 1:2 whole blood/normal saline mixture on Lymphocyte Separation Media (in 1.5:1 ratio, MP Biomedicals 0850494X) and centrifuging at 500x*g* for 10 minutes and then collecting the buffy coat. The buffy coat cells were then pelleted and used for downstream analysis. Transfection was performed using Lipofectamine 2000 (Invitrogen) as described in the manufacturer's protocol. Normal growth medium was replaced 12-16 hours after transfection. Custom gapmeRs targeted to *SNHG12* (Qiagen) or a negative control (Qiagen) and siRNA targeting IMP3(Origene, SR307255), YBX1 (Origene, SR303243), DHX9 (Origene, SR301175), and DNA-PK (OriGene, SR321433) were used for transfection. For murine in vivo gapmeR injections, 37.5 nmol naked gapmeR targeted to Snhg12 or control (Qiagen, LG00166878-DFA and LG00000002) was injected in 100 µl volume by tail vein injection with two-day loading protocol followed by biweekly injections until sacrifice. For intramuscular gastrocnemius gapmeR delivery in *db/db* mice, 37.5 nmol gapmeR was mixed with 25 µl Lipofectamine 2000 in 100 µl OptiMEM according to manufacturer's protocol and injected at three points (distal, mid and proximal gastrocnemius muscle).

Co-immunoprecipitation assay (Co-IP)

Co-IP assays were performed as described in *Haemmig S et. al.* 2020. Antibodies used for Co-IP assays included those against DNA-PK (1 µg antibody / 200 µg lysate, Abcam, ab702501), DHX9 (Abcam, 1 µg antibody / 200 µg lysate, ab26271), YBX-1 (1 µg antibody / 200 µg lysate, Cell Signaling 9744S), IMP3 (1 µg antibody / 200 µg lysate, Abcam ab177477).

Skeletal muscle histology

Skeletal myofiber cross sectional area (CSA) was assessed by immunofluorescence microscopy. Gastrocnemius muscles were sectioned, deparaffinized and rehydrated. Antigen retrieval was performed using an antigen unmasking solution (Vector Laboratories, H-3300). Muscle sections were then permeabilized with 0.25% Triton X-100 (ThermoScientific, 28313) for 10 minutes followed by three, two-minute washes in PBS. Sections were then

blocked in PBS supplemented with 1% BSA and 5% goat serum for 1 hour at room temperature. The slides were then briefly washed with PBS and incubated with 5µg/µl wheat germ agglutin (ThermoScientific, W32466) for 30 minutes to label myofiber membranes. Coverslips were mounted with Vectashield hardmount containing DAPI (Vector Laboratories, H-1500). Images were obtained at 20x magnification using an Evos FL2 Auto microscope (ThermoScientific) and tiled images of the entire muscle section were used for analysis. Non-myofiber area was quantified by thresholding tiled images to obtain the pixel area of tissue between myofibers stained by wheat germ agglutin. Myofiber CSA were analyzed using MuscleJ (69), an automated image analysis software developed within Fiji.

Immunofluorescence

For immunofluorescence staining, gastrocnemius muscle isolated from mice was fixed in 4% PFA (Boston Bio Products) for 24-48 hours and embedded in paraffin and sectioned for staining. Slides were cut, deparaffinized, and hydrated. Antigen retrieval was performed by boiling the slides for 10 minutes in either Sodium Citrate pH 6 or Tris-EDTA pH9 with a pressure cooker. The sections were blocked with 5% donkey serum (Jackson ImmunoResearch Lab) for an hour at room temperature. Slides were then incubated with rat anti-CD45 (1:200, Novus Biological, NB100-77417), rabbit anti-CD206 (1:250, Abcam, ab64693), anti-CD31 (1:50, Dianova, DIA310), mouse anti-SMA (1:500, Sigma Aldrich, A5228), rabbit anti-gamma H2AX (1:500, Abcam, ab11174), rabbit anti-CCR7 (1:500, Abcam, ab32527) overnight at 4°C. The slides were washed three times in TBS and incubated with the following secondary antibodies (all from Jackson ImmunoResearch Lab): (1) Cy3 conjugated donkey anti-rat secondary antibody (1:300, Cat#: 712-165-153) and Alexa 647 conjugated donkey anti-rabbit secondary antibody (1:300, Cat#: 711-605-152) and Alexa 488 conjugated donkey anti-rabbit secondary antibody (1:300, Cat#: 711-545-152); (2) Alexa 647 conjugated Donkey anti-rabbit secondary antibody (1:300, Cat#: 711-605-152), CY3 conjugated donkey anti-rat secondary antibodies (1:300, Cat#: 712-165-153) and Alexa 488 conjugated donkey anti-mouse secondary antibodies (1:300, Cat#: 715-545-151); (3 and 4) Cy3 conjugated donkey anti-rabbit secondary antibody (1:300, Cat#: 711-165-152) and Alexa 647 conjugated donkey anti-rabbit secondary antibody (1:300, Cat#: 711-605-152) for 90 minutes at room temperature. After secondary antibody incubation, the tissue sections were counterstained with Hoechst 33342 (Invitrogen) for nuclear staining. The tissue sections were then washed three times with TBS and the slides were mounted with Prolong Gold anti-fade mounting media (Invitrogen). This IF staining was performed by BIDMC confocal imaging and IHC core facility. Images were

acquired on a Carl Zeiss LSM 880 confocal microscope using Zen black software version 2.3 SP1. Objective lenses 10x 0.45 NA and 20x 0.8 NA were used for image acquisition. The data were calculated from tile scans from groups of 6-9 mice. For each mouse, 2-5 regions of interest were randomly selected for imaging and used for quantitation.

Lentivirus production and transduction

Lentivirus for pUltra (Malcolm Moore, Addgene, 24129) was generated by co-transfection of 293T cells (ATCC) using Lipofectamine 3000 (Life Technologies) with pMD2.G (Didier Trono, Addgene) and psPAX2 (Didier Trono, Addgene) in a ratio 3:2:1, respectively. Transfection mix was added dropwise to dish and medium was changed 16hrs later. The supernatant was collected two days later by filtering through 0.45µm filter and stored at -80°C in 5 ml aliquots. Transduction of HUVECs was carried out in 6-well, 12-well, or 24 well plates by adding 1:1 lentiviral supernatant/medium in combination with 8 µg/mL polybrene (American Bio). Normal growth medium was replaced after 36 hours.

LncRNA pulldown

Biotinylated RNA was generated using T7 RNA polymerase kit (Thermo Scientific) by adding 1 µg linearized plasmid DNA, 10X biotin RNA labeling mix (Roche, 11685597910), 5x transcription buffer (Agilent) and RNase-free water in total volume of 20 µl and incubated for 2 hours at 37°C. Subsequently, DNase I (NEB) was added and the reaction was further incubated 15 min at 37°C to remove DNA template. The reaction was terminated using 0.8 µl 0.5M EDTA (pH 8.0). Purified biotinylated RNA was obtained using G-50 Sephadex Columns (Sigma Aldrich, 112739965001) according to the manufacturer's protocol. After RNA concentration was determined on NanoDrop (Thermo Fisher), the purified RNA was immediately used, or stored at -80°C. When needed, 10 pmol of biotinylated RNA (calculated using https://www.promega.com/resources/tools/biomath/) was heated for 2 min at 90°C in RNA structure buffer (10 mM Tris pH 7.0, 0.1 M KCl, 10 mM $MgCl_2$). The mix was immediately transferred to ice and incubated 2 min and subsequently incubated at room temperature for 20 min.

For in vitro Co-IP experiments, nuclear pellets were prepared by resuspending 10^7 HUVECs in 2:2:6 mL mixture of (PBS/nuclear isolation buffer (1.28 M sucrose, 40 mM Tris-HCl pH 7.4, 20 mM $MgCl_2$, 4% Triton X-100)/RNase-free water) on ice and incubated for 20 minutes with disruption every 4-5 minutes. Pellets of nuclei were harvested at 2,500x*g* for 15 min and the pellet was resuspending in 1 mL RIP buffer (0.15 M KCl, 25 mM Tris-HCl pH 7.4, 5 mM EDTA, 0.5 % NP-40, 0.5mM DTT (Sigma), 100 U/mL RNase inhibitor (Invitrogen), 1x

protease inhibitor cocktail (Roche, 11836153001)). Nuclei were homogenized by 18 strokes using a 1-1.5 mL dounce homogenizer, followed by centrifugation at 15,000xg for 15 min. The supernatant containing nuclear protein was transferred to a new tube and pre-cleared by applying 60 µl of Streptavidin agarose beads (Thermo Scientific) for 1 hour at 4°C. After clearing, 10 pmol of properly folded biotinylated RNA and 1 µg/µL yeast tRNA (Ambion, AM7119) were added into the pre-cleared nuclear lysate (200 µg) and incubated 2 hours at 4°C. Beads were then collected by centrifugation at 12,000x*g* and washed with 1 mL ice-cold NT2 buffer (50mM Tris-HCl pH 7.4, 0.15 M NaCl, 1m M $MgCl_2$, 0.05 % NP-40, 100U/mL RNase inhibitor, 400nM Vanadyl-ribonucleoside complex (BioLabs), 1x protease inhibitor cocktail) at 4°C five times. After washing, proteins were denaturated in 40 µL 2×Laemmli loading buffer (4% SDS, 120 mM Tris-HCl pH 6.8, 0.02% bromophenol blue, 0.2 M DTT) at 98°C for 10 min for subsequent immunoblotting.

For in vivo pulldowns of Snhg12-interacting proteins, biotinylated RNA was injected on consecutive days 1 and 2 by tail vein (15 µg/injection) before aortas were isolated on day 3. The tissue was processed as described above for cell lysate and nuclei were isolated using a Nuclear Extraction Kit (Millipore).

RNA synthesis, modification and injection

10 µg linearized and purified T7 vector with the cassette for LacZ or SNHG12 was used for 1x T7 RNA polymerase transcription reaction (Promega, RiboMax Large Scale RNA, PRP1300) based on the manufacturer's protocol. After 4 hours at 37°C, RNA was purified with phenol:chloroform isolation and resuspended in 140 µl RNase-free water. After 5 minutes at 65°C, RNA was capped and 2'-*O*-methylated using Vaccinia Capping System (Fisher 50591120 and NEB M2080S) based on manufacturer's protocol before purification on Qiagen Universal Midi Kit RNeasy columns. RNA was stored at -80°C prior to use.

Clinical RNA-Seq Analysis

Using the published database by Ryan et. al. (accession number GSE120642) obtained from the GEO database through PubMed, the normalized counts file was used to generate relative expression dot-plot graphs with mean and standard error for various genes of interest that are involved in the angiogenesis response. Comparisons were made between healthy adult (HA), ischemic claudicant (IC) and critical limb ischemia (CLI) groups, by normalizing to the HA (at 100%) as a control cohort. Statistical comparisons were made using 1-way ANOVA. Data are displayed as relative expression.

RNA Isolation and RT-qPCR

Tissues (liver, gastroc) were homogenized using 5mm Stainless steel beads (Qiagen) TissueLyser II (Qiagen) according to manufacturer's instruction. For RNA isolation, TRIzol reagent (Invitrogen) or RNeasy kit (Qiagen) was used based on manufacturer's protocol. Subsequent RT-qPCR was performed using High-Capacity cDNA Reverse Transcription kit (Applied Biosystems). For SyberGreen based assay GoTaq qPCR Master Mix (Promega) was used. Expression of mRNAs and lncRNA expression levels were normalized to HPRT, or β-actin (Aglient, AriaMx Real Time PCR System). Changes in expression were calculated using delta-delta Ct method.

RNA immunoprecipitation (RIP)

Nuclear pellets were prepared, harvested and homogenized by resuspending a pellet of 10^7 HUVECs in nuclear isolation buffer as noted above in LncRNA pulldown methods section. After homogenization, the supernatant was cleared by applying 40 µg magnetic protein A/G beads (Thermo Scientific) with non-specific rabbit IgG antibody (Invitrogen). The supernatant was collected and 50µL lysate was saved as an input control. The remaining lysate was divided into multiple portions of equal volume and 1 µg of protein-specific antibody was added and samples were incubated for 1 hour at 4°C in the presence of fresh magnetic protein A/G beads. Protein-specific antibodies used included IMP3 (Cell Signaling 57154S), YBX1 (Cell Signaling 4202S), DHX9 (Abcam ab26271), and DNA-PKcs (Abcam, 70250) or no antibody. Immuno-complexes captured by magnetic beds were washed three times with ice-cold RIP buffer and resuspended in Trizol (Invitrogen). RT-PCR was subsequently performed using the same input volumes.

Western blot

Proteins were isolated using RIPA buffer (Boston BioProducts) with protease inhibitor cocktail (Roche) and phosphatase inhibitors (Invitrogen). Protein concentrations were determined using Pierce BCA assay (Thermo Scientific). 15 µg protein were loaded per lane on a 4-20% Mini-PROTEAN TGX Gel (Bio-Rad). Separated proteins were transferred to PVDF membranes using the Transfer Turbo Blot system (Bio-Rad) and Trans-Blot Turbo RTA Transfer Kit (Bio-Rad, 170-4272). The membrane was blocked with 5% non-fat milk in TBST for 1h at room temperature. After blocking, the membrane was incubated overnight at 4 °C with antibodies against □H2AX

(Cell Signaling, 9718, 1:1000), DNA-PK (Abcam, ab32566, 1:1000), DHX9 (Abcam, ab26271, 1:1000), YBX-1 (Cell Signaling, 4202S, 1:1000), IMP3 (Cell Signaling, 57145S, 1:1000), hnRNP A1 (Cell Signaling, 8443S, 1:1000), hnRNP K (Abcam, ab52600, 1:1000), DDX3Y (Fisher, NBP132780, 1:1000), PRKCDBP (Fisher, PIPA519187, 1:1000), histone H3 (Cell Signaling, 14269S, 1:1000), vinculin (Cell Signaling, 4650S, 1:1000), and GAPDH (Cell Signaling, 2118, 1:4000). Secondary antibodies used were anti-mouse HRP-linked IgG (Cell Signaling, 7076S, 1:5000) or anti-rabbit HRP-linked IgG (Cell Signaling, 7074S, 1:5000). Quantification and visualization of protein bands was performed using SuperSignal West Femto Maximum Sensitivity Substrate (Life Technologies) and a luminescent image analyzer (BioRad, Chemidoc).

RNA-Seq analysis

RNA-Seq analysis was performed after ribodepletion and standard library construction using Illumina HiSeq2500 V4 2x100 PE (Genewiz). All samples were processed using an RNA-seq pipeline implemented in the bcbio-nextgen project (https://bcbionextgen.readthedocs.org/en/latest/). Raw reads were examined for quality issues using FastQC (http://www.bioinformatics.babraham.ac.uk/projects/fastqc/) to ensure library generation and sequencing were suitable for further analysis. Trimmed reads were aligned to UCSC build mm10 of the mouse genome and augmented with transcript information from Ensembl releases 86 (H. sapiens) and 79 (M. musculus) using STAR (60). Alignments were checked for evenness of coverage, rRNA content, genomic alignment context and other quality checks using a combination of FastQC and Qualimap (61). Counts of reads aligning to known genes were generated by featureCounts (62). Differential expression at the gene level was called with DESeq2 (63). Total gene hit counts and CPM values were calculated for each gene and downstream differential expression analysis between specified groups was performed using DESeq2 and an adapted DESeq2 algorithm that excludes overlapping reads. Genes with adjusted FDR< 0.05 and log2fold-change (>1.5) were called as differentially expressed genes for each comparison. The mean quality score of all samples was 35.91 with a range of 28,000,000-63,000,000 reads per sample. All samples had at least >96% of mapped fragments over total fragments. Coverage was visualized using Integrative Genomics Viewer (IGV) (version 2.3.68). Ingenuity Pathway Analysis/IPA (QIAGEN) and MetaCore (v20.2) were used for functional/gene set enrichment analysis. RNA-seq data will be available through the Gene Expression Omnibus upon publication.

Figure 1S: *In vitro* angiogenesis assays using SNHG12 gain- and loss-of-function models.

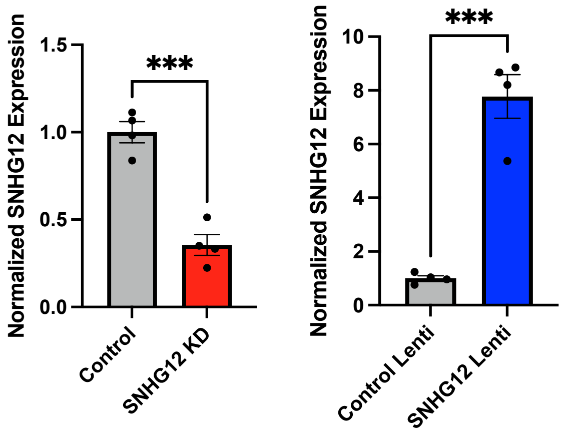

Figure 1S: SNHG12 expression in gain- and loss-of-function models by qRT-PCR. Cells treated as those in Figure 2 were isolated at 48 hours after transfection or transduction with either control or SNHG12 gapmeR or lentivirus. SNHG12 expression relative to HPRT RNA was measured by qRT-PCR by the method of delta-delta Ct. (n=4 per condition) ***$P<0.001$ using Student's t-test.

Figure 2S: Knockdown of SNHG12 *in vivo* in a C57BL/6 hind-limb ischemia model.

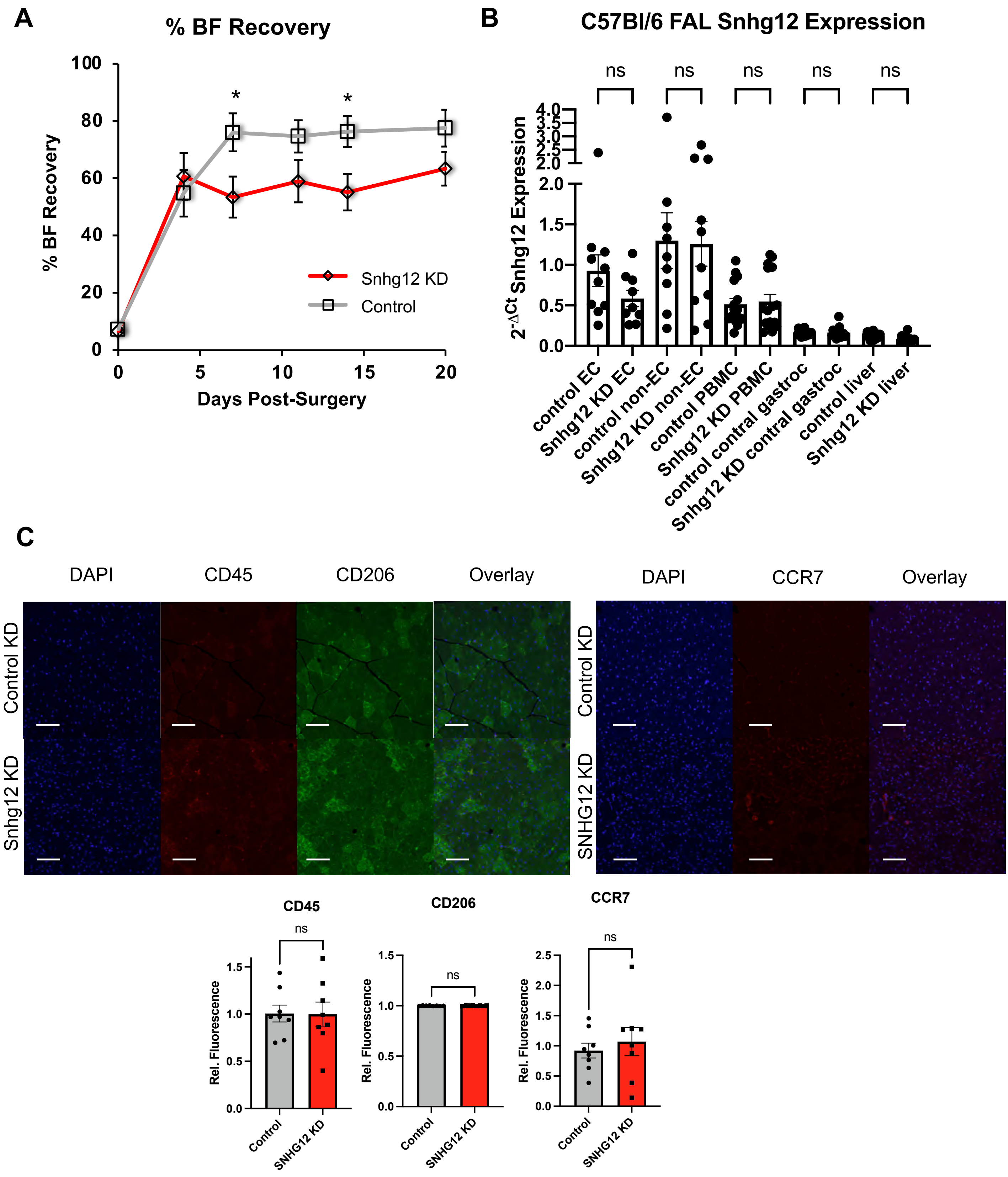

Figure 2S: Knockdown of Snhg12 *in vivo* in a C57BL/6 hindlimb ischemia model. (A) Percent BF recovery after hindlimb ischemia is plotted over a time-course of 20 days after femoral artery ligation. **(B)** SNHG12 expression in gastrocnemius EC and non-EC ractions, PBMCs, contralateral gastrocnemius, and liver from Snhg12- versus control gapmeR-injected mice (n=10-16 per group). Snhg12 expression shows a trend towards decreased expression. **(C)** Microscopy of gastrocnemius muscle shows no increase in $CD45^+$ cell infiltration and no changes in $CD206^+$ (M2 marker) or $CCR7^+$ (M1 marker) macrophages. Quantification performed on 3-5 sections per gastrocnemius, n=16 mice per group, scale bar = 50 μm). * P<0.05 using Student's t-test.

Figure 3S: Muscle fiber staining shows increased damage in SNHG12 KD FAL C57Bl/6 mice.

A

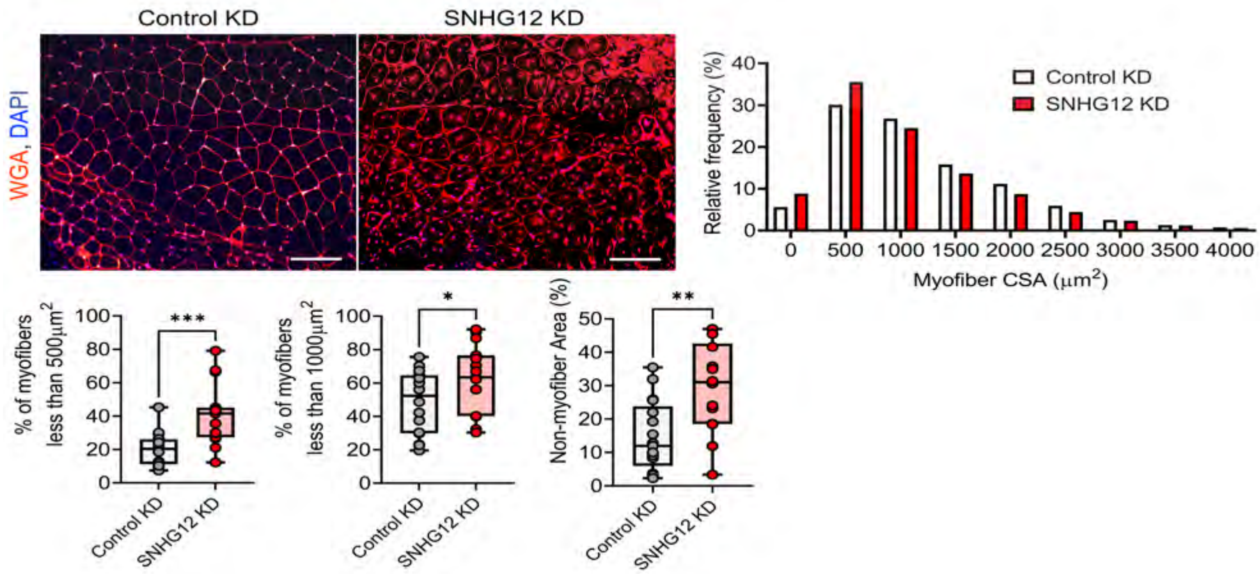

B

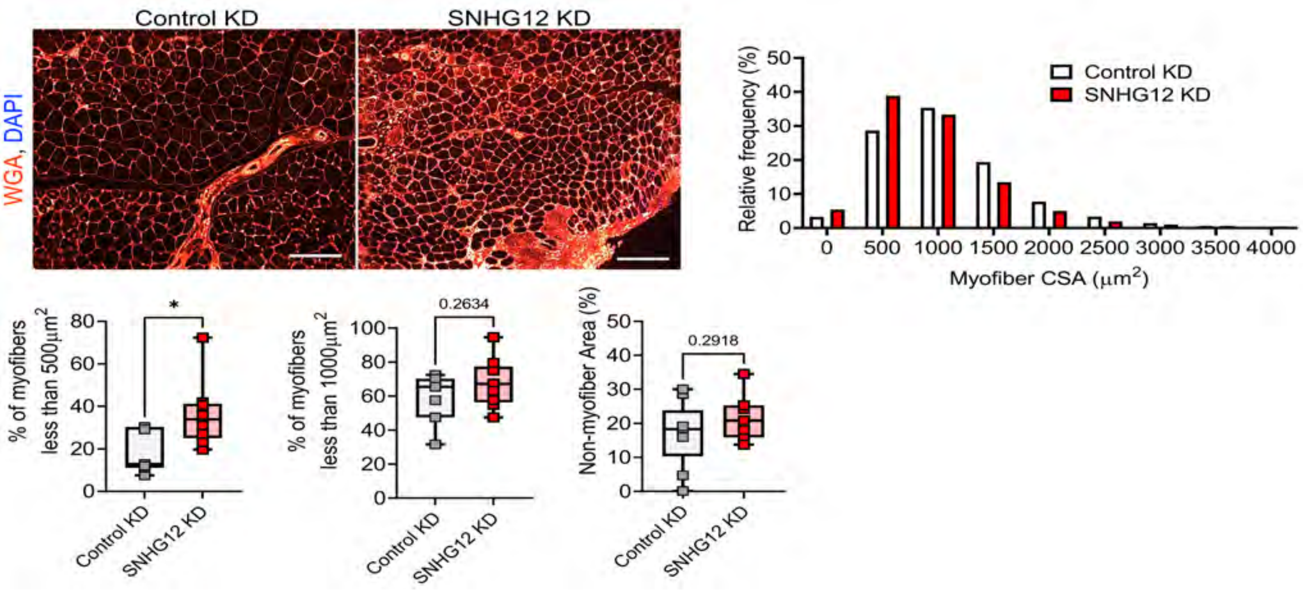

Figure 3S: Muscle fiber staining shows a leftward shift to smaller myofiber size in SNHG12 KD mice. A. Microscopy images of gastrocnemius muscle labeled with wheat germ agglutin (WGA) and DAPI show an increase in the percentage of small myofibers less than 500µm^2 or 1000µm^2 and expansion of non-myofiber area, features demonstrating increased ischemic myopathy in SNHG12 KD-treated C57BL6 mice. Scale bars = 200µm. *P<0.05, **P<0.01, ***P<0.001 using two-tailed *t*-test. * P<0.05 using Student's t test. **B.** Microscopy images of gastrocnemius muscle labeled with wheat germ agglutin (WGA) and DAPI show an increase in the percentage of small myofibers less than 500µm^2 and non-significant trend in expansion of non-myofiber area, features demonstrating increased ischemic myopathy in SNHG12 KD-treated *db-db* mice. Scale bars = 200µm. *P<0.05 using two-tailed *t*-test.

Figure 4S: Tissue SNHG12 Expression Prior to Femoral Artery Ligation.

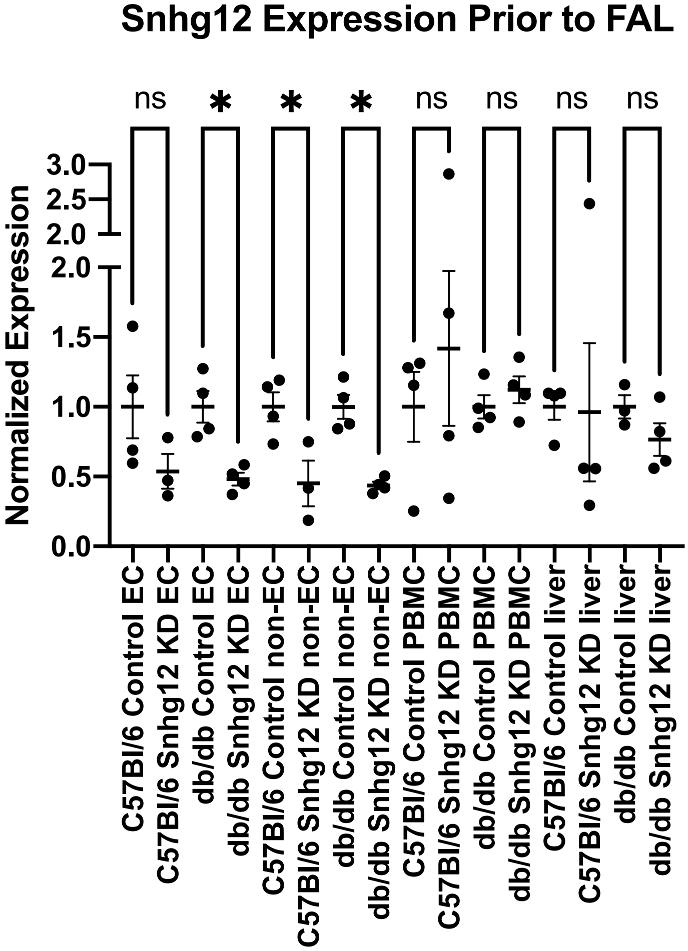

Figure 4S: Tissue Snhg12 Expression Prior to Femoral Artery Ligation. C57Bl/6 or *db/db* mice were injected with gapmeRs on day 1 and day 2 and sacrificed on day 3. Liver, PBMCs, and gastrocnemius EC and non-EC fractions were isolated and RNA was purified for qRT-PCR analysis of Snhg12 compared to Hprt, which is normalized for each tissue. There is significant decrease in the expression of Snhg12 compared to Hprt in the EC and non-EC fractions of gastrocnemius muscle in *db/db* and non-EC of C57Bl/6 and a trend in EC from C57Bl/6. * P<0.05 using Student's t test.

Figure 5S: Expression of SNHG12 in a BALB/c hindlimb ischemia model.

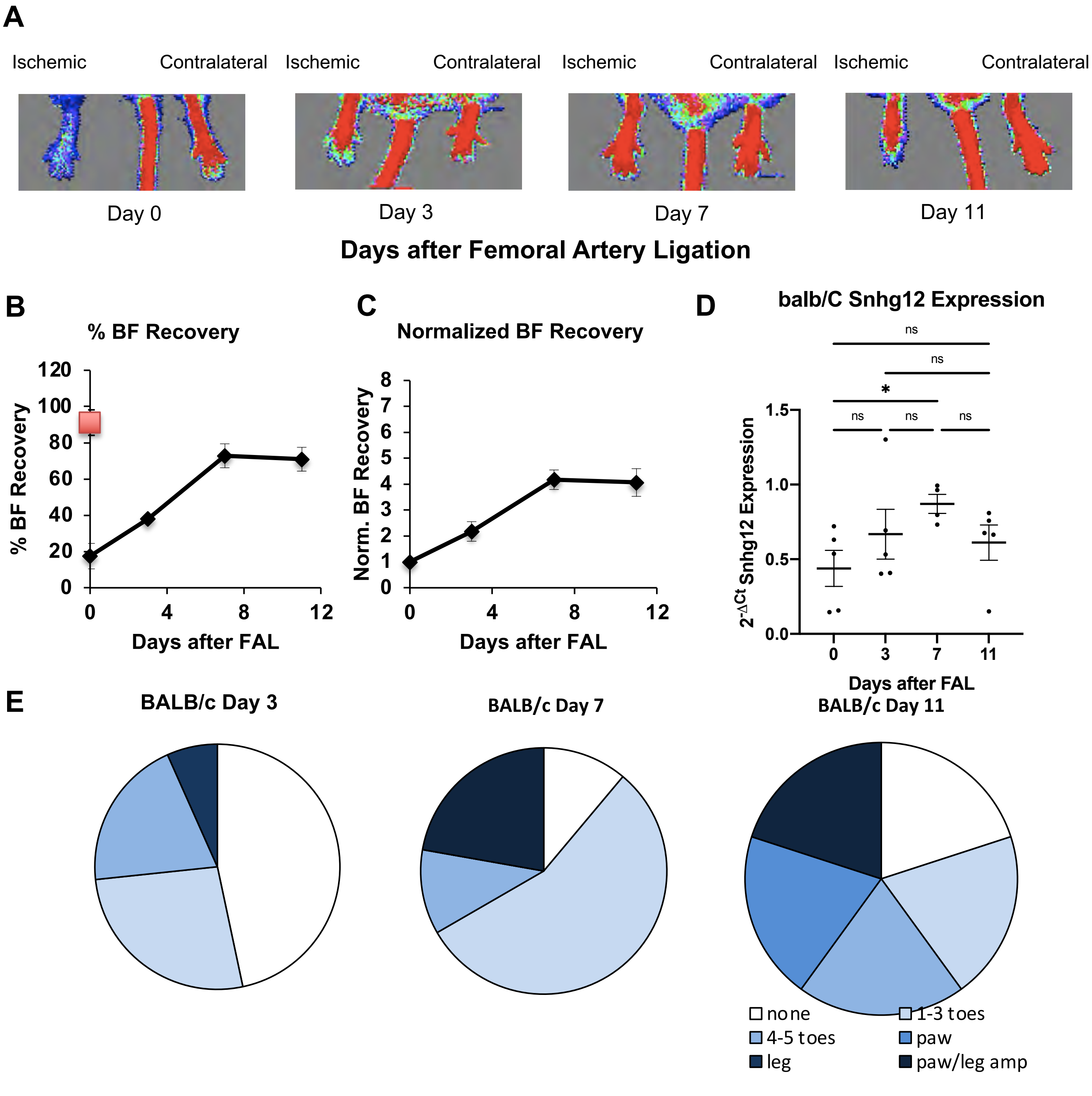

Figure 5S: Expression of SNHG12 in a BALB/c hindlimb ischemia model. Snhg12 is dysregulated in a mouse model of acute hindlimb ischemia and in human cohorts with critical limb ischemia. **(A)** Anesthetized mice that underwent sham surgery (n=5) or femoral artery ligation (n=15), were imaged by laser Doppler imaging in the supine position to evaluate blood flow (BF) in the ischemic limb versus contralateral limb on days 0, 3, 7, and 11 (representative images shown at these time points). **(B)** Laser Doppler imaging results were quantified using MoorLDI software in order to show fold increase in BF compared to the immediate post-operative ischemic BF (normalized BF recovery). **(C)** Mice were sacrificed at day 0 (n=5), day 3 (n=5), day 7 (n=5), and day 11 (n=5) and RNA was isolated from gastrocnemius muscle and qRT-PCR was performed for Snhg12 compared to Hprt control. * P<0.05 using 1-way ANOVA.

Figure 6S: Knockdown of Snhg12 in a BALB/c hind-limb ischemia model.

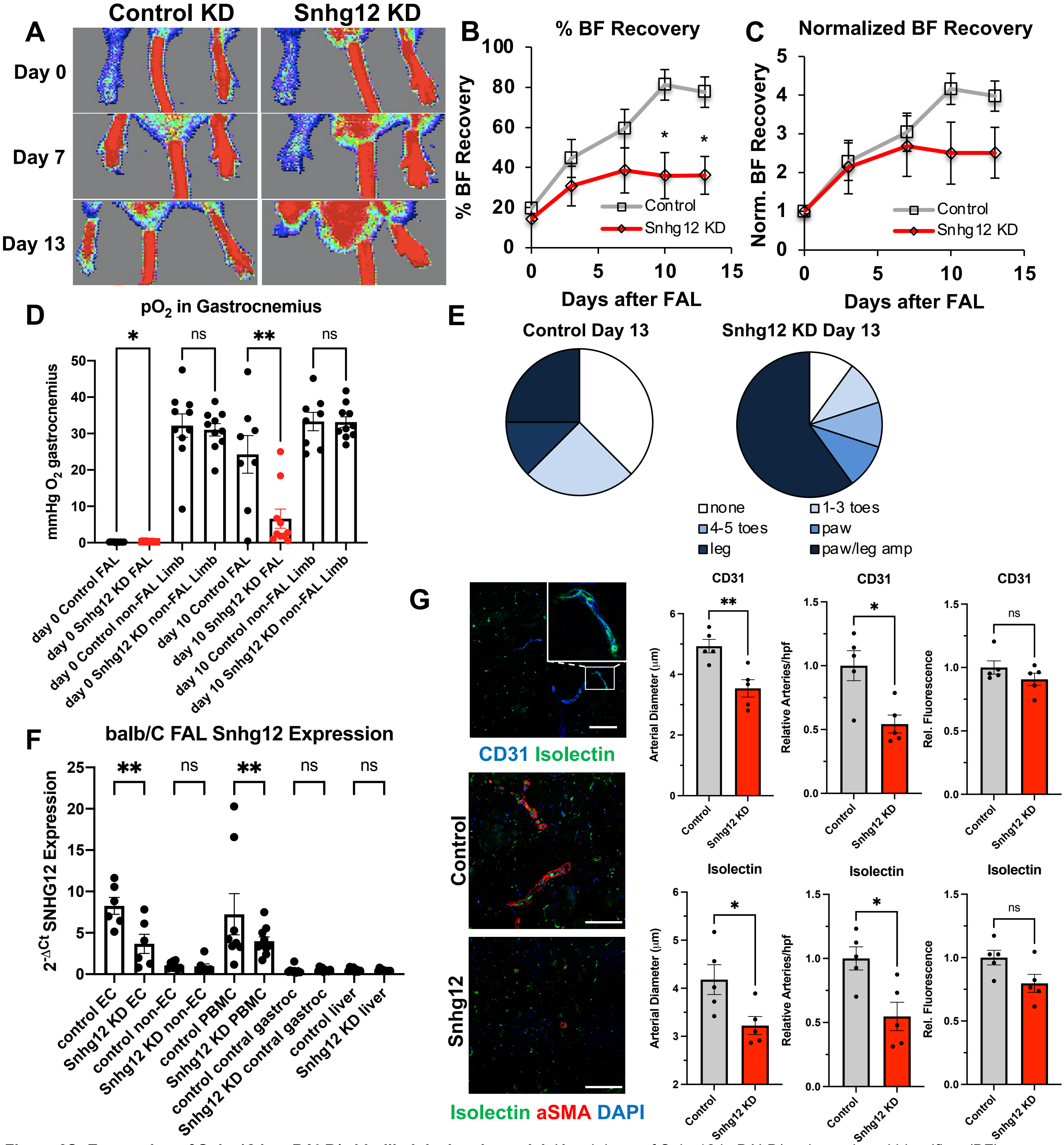

Figure 6S: Expression of Snhg12 in a BALB/c hindlimb ischemia model. Knockdown of Snhg12 in BALB/c mice reduced bloodflow (BF) recovery after femoral artery ligation (FAL). **(A)** Mice that were intramuscularly injected with control or Snhg12 gapmeRs were subjected to FAL as shown in the schematic (left panel). Laser Doppler imaging was performed on mice (n=10 in each group) with representative images from days 0 and 13 shown (right panel). **(B)** Percent BF recovery is shown for Snhg12 gapmeR-injected mice versus control gapmeR-injected mice. **(C)** Normalized BF Recovery is shown for respective groups of mice (n=6-10 per group). **(D)** Oxygen concentrations at day 0 and 10 after FAL in ischemic and contralateral gastrocnemius muscle from Snhg12 - versus control gapmeR-injected mice. Non-FAL limbs were untreated with gapmer (n=6-10 per group). **(E)** Ischemia scores for toes, paws, and leg show a trend for more areas of limb necrosis in the Snhg12 gapmeR knockdown group. **(F)** Snhg12 expression in gastrocnemius, PBMCs, and liver from Snhg12 versus control gapmeR-injected mice (n=6-8 per group). **(G)** Microscopy of fixed and sectioned gastrocnemius muscle from mice sacrificed on day 13 after systemic injection of FITC-lectin. CD31 and isolectin co-staining of vessels occurs in overlapping fashion in gastrocnemius. Control versus SNHG12 gapmeR-injected mice revealed a 23-28% reduction in average arterial diameter (by CD31 or isolectin, respectively) and a 46% reduction in the number of arteries per high-powered field (hpf) but no overall change in total CD31 or isolectin staining (5 sections per gastrocnemius, n=5 mice per group, scale bar = 100 μm). * $P<0.05$, ** $P<0.01$ using Student's t-test.

Figure 7S: Table of SNHG12-interacting proteins identified by mass spectrometry.

A

Accession Number	Protein Description	# Hits SNHG12	# Hits LacZ
gi|4506741|ref|NP_001002.1|	40S ribosomal protein S7 [Homo sapiens]	4	0
**gi|13654237|ref|NP_008835.5|**	**DNA-dependent protein kinase catalytic subunit isoform 1 [Homo sapiens]**	**4**	**0**
gi|14165469|ref|NP_001010.2|	40S ribosomal protein S15a [Homo sapiens]	4	1
gi|15055539|ref|NP_002943.2|	40S ribosomal protein S2 [Homo sapiens]	4	1
**gi|100913206|ref|NP_001348.2|**	**ATP-dependent RNA helicase A [Homo sapiens]**	**4**	**1**
**gi|13514809|ref|NP_004651.2|**	**ATP-dependent RNA helicase DDX3Y [Homo sapiens]**	**4**	**1**
**gi|4504445|ref|NP_002127.1|**	**heterogeneous nuclear ribonucleoprotein A1 isoform a [Homo sapiens]**	**4**	**1**
**gi|14165435|ref|NP_112552.1|**	**heterogeneous nuclear ribonucleoprotein K isoform b [Homo sapiens]**	**4**	**1**
gi|119703753|ref|NP_005546.2|	keratin, type II cytoskeletal 6B [Homo sapiens]	4	1
**gi|34098946|ref|NP_004550.2|**	**nuclease-sensitive element-binding protein 1 [Homo sapiens]**	**4**	**1**
gi|4506649|ref|NP_000958.1|	60S ribosomal protein L3 isoform a [Homo sapiens]	4	2
gi|16753227|ref|NP_000961.2|	60S ribosomal protein L6 [Homo sapiens]	4	2
**gi|30795212|ref|NP_006538.2|**	**insulin-like growth factor 2 mRNA-binding protein 3 [Homo sapiens]**	**4**	**2**
gi|4506605|ref|NP_000969.1|	60S ribosomal protein L23 [Homo sapiens]	4	3
gi|4506623|ref|NP_000979.1|	60S ribosomal protein L27 [Homo sapiens]	4	3
**gi|4504301|ref|NP_003529.1|**	**histone H4 [Homo sapiens]**	**4**	**3**
**gi|47132587|ref|NP_659477.2|**	**protein kinase C delta-binding protein [Homo sapiens]**	**4**	**3**

Bold = non-ribosomal, non-keratin proteins

Figure 7S: Table of SNHG12-interacting proteins identified by mass spectrometry. Accession numbers and protein descriptions are arranged in descending specificity for SNHG12 interaction compared to lacZ. Boldened are the non-ribosomal, non-keratin proteins.

Figure 8S: Angiogenesis Functional Assays of SNHG12-Interacting Proteins.

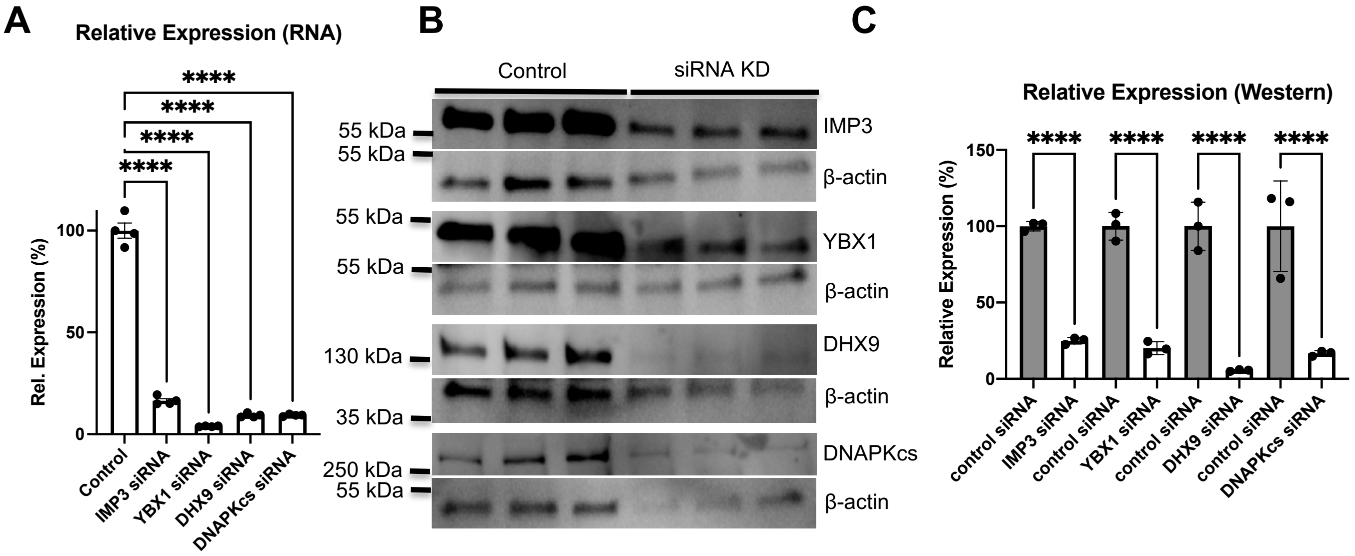

Figure 8S: Expression of SNHG12-interacting proteins in angiogenesis assays. (left panel) Quantitative RT-PCR shows that siRNA-mediated knockdown of IMP3, YBX1, DHX9, or DNAPK in HUVECs yields 80-95% knockdown at 60-72 hours after transfection (n=4 per condition). (middle panel) Western blot of HUVECs transfected with siRNAs against IMP3, YBX1, DHX9, or DNAPK shows 75-95% decreased protein expression three days after transfection (quantified by densitometry on right panel) (n=3 per siRNA). **** $P<0.0001$ by Student's t test or one-way ANOVA.

Figure 9S: Knockdown of SNHG12 *in vivo* in a diabetic hind-limb ischemia model.

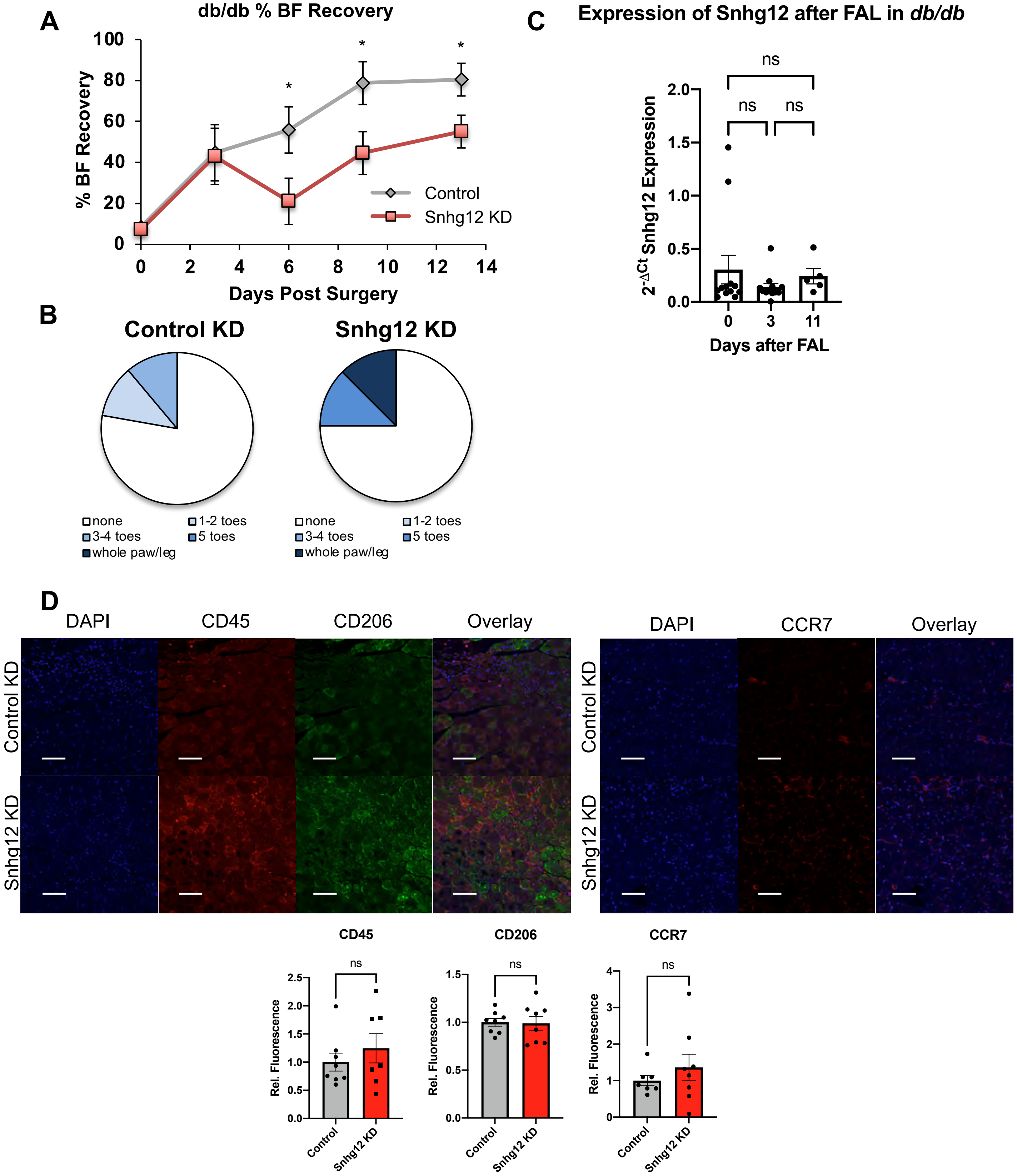

Figure 9S: Knockdown of Snhg12 in a diabetic hindlimb ischemia model. (A) Percent BF recovery after hindlimb ischemia in *db/db* mice injected with Snhg12 or control gapmeR into gasctrocnemius is plotted over a time-course of 13 days after femoral artery ligation (n=8-9 per condition). **(B)** Ischemia scores for toes, paws, and leg show a trend for more areas of limb necrosis in the Snhg12 gapmeR knockdown group (n=8-9 per condition). **(C)** Gastrocnemius Snhg12 expression after FAL in *db/db* mice shows similar kinetics compared to C57Bl/6 mice (n=5-12 per condition). **(D)** Microscopy of gastrocnemius muscle shows no change in CD45⁺ cell infiltration and no changes in CD206⁺ (M2 marker) or CCR7⁺ (M1 marker) macrophages invasion. Quantification performed on 6-9 fields per gastrocnemius, n=8-9 mice per group, scale bar = 50 μm). * P<0.05 using Student's t-test or 1-way ANOVA.

Figure 10S: Volcano plot of SNHG12 knockdown in HUVECs.

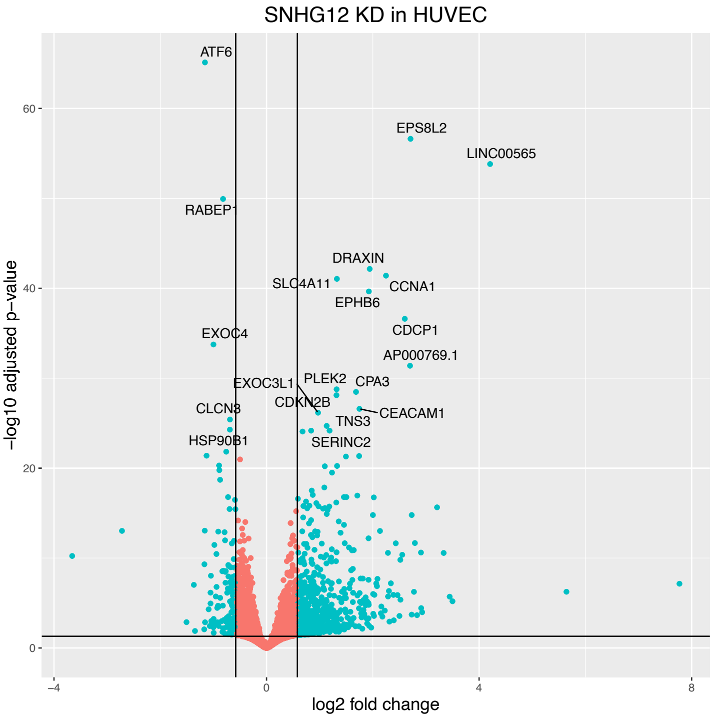

Figure 10S: Volcano plot of SNHG12 knockdown in HUVECs. Top 20 most significant differentially expressed genes are labeled.

Figure 11S: MetaCore Analysis of SNHG12-interacting proteins YBX1, DHX9 and DNAPK.

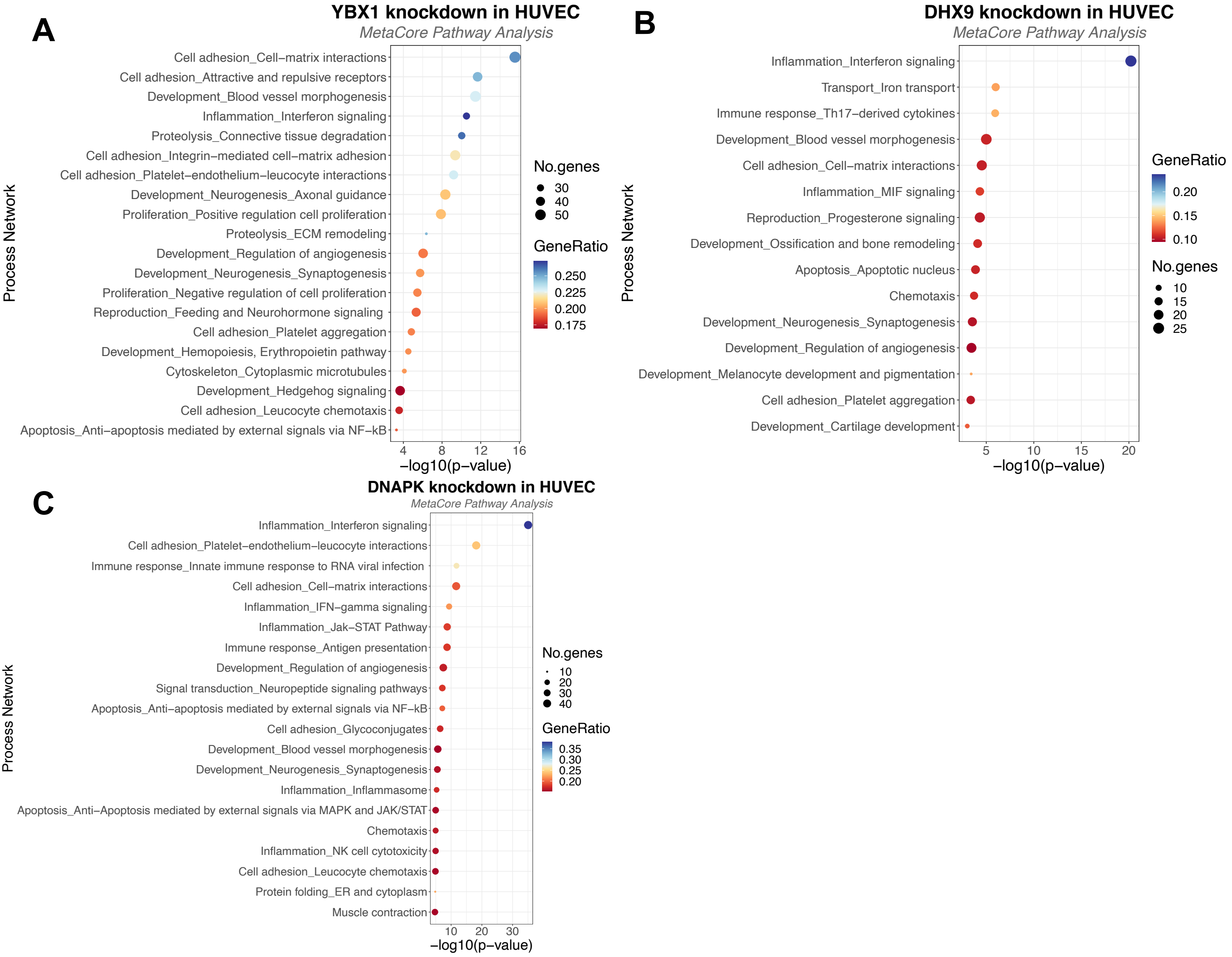

Figure 11S: MetaCore Analysis of SNHG12-interacting proteins YBX1, DHX9, and DNAPK. Gene set enrichment analysis using MetaCore process networks for HUVECs knocked down for YBX1, DHX9 and DNAPK (n=4 per group for RNA-seq compared to control) showing top 20 significant process networks (only 15 networks were significant in the case of DHX9).

Figure 12S: RNAseq analysis of SNHG12 knockdown in *db/db* endothelial cells after FAL.

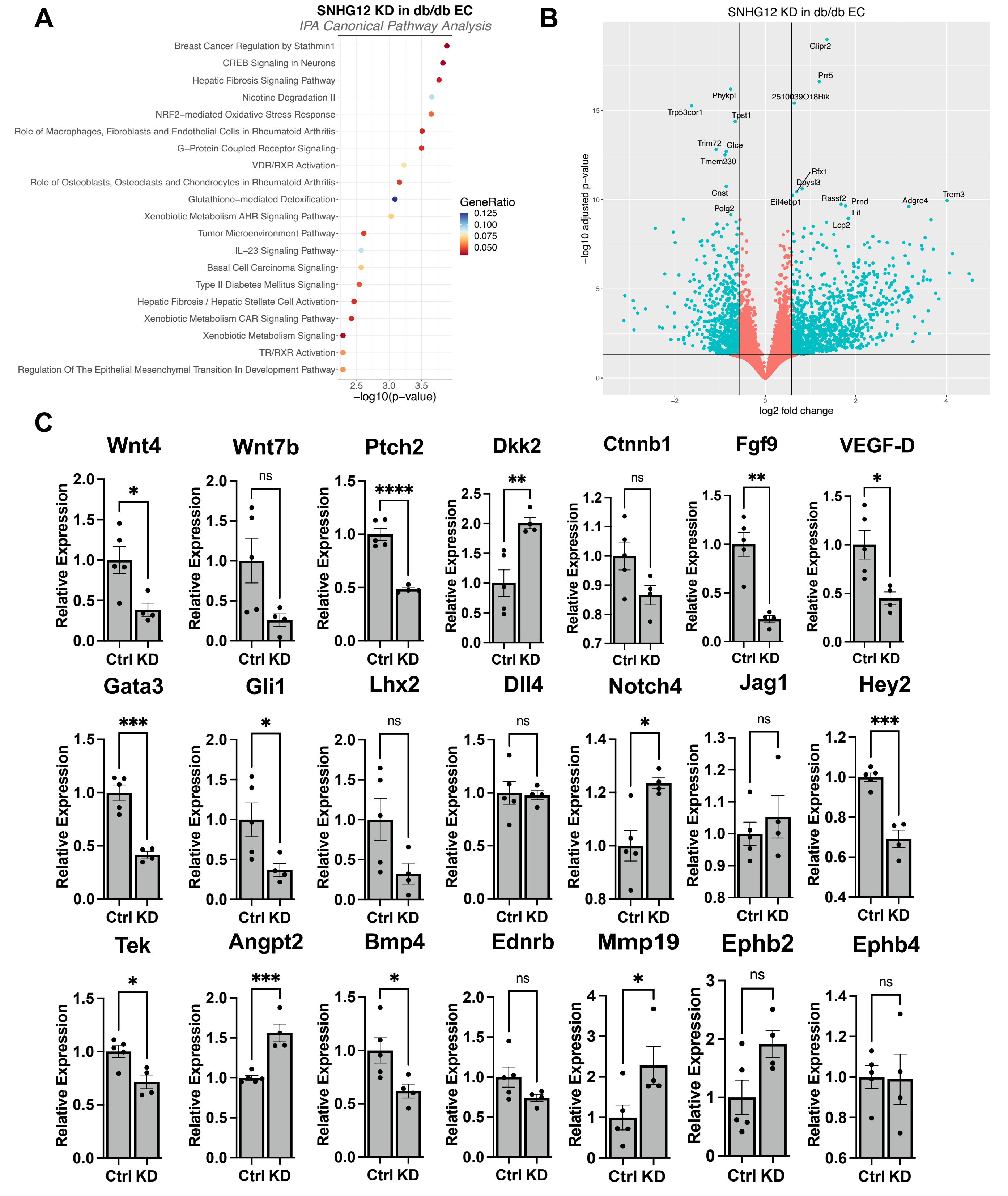

Figure 12S: RNA-seq analysis of Snhg12 knockdown in *db/db* endothelial cells after FAL. (A) Ingenuity pathway analysis reveals various pathways that heavily represent Notch, Wnt, Bmp and angiopoietin signaling pathway elements. **(B)** Volcano plot of most significant differentially expressed genes. **(C)** Selected genes from Notch, Wnt, Angiopoietin, Bmp, Endothelin, Ephrin pathways plotted for relative expression from RNA-seq data show a downregulation of various Wnt elements, upregulation of Wnt inhibitor Dkk2 and anti-angiogenic Mmp19 and downregulation of Fgf9, Vegf-D and several proangiogenic transcription factors. * P<0.05, ** P<0.01, *** P<0.001, **** P<0.0001 using Student's t-test.

Figure 13S: RNAseq analysis of SNHG12 knockdown in *db/db* non-ECs after FAL.

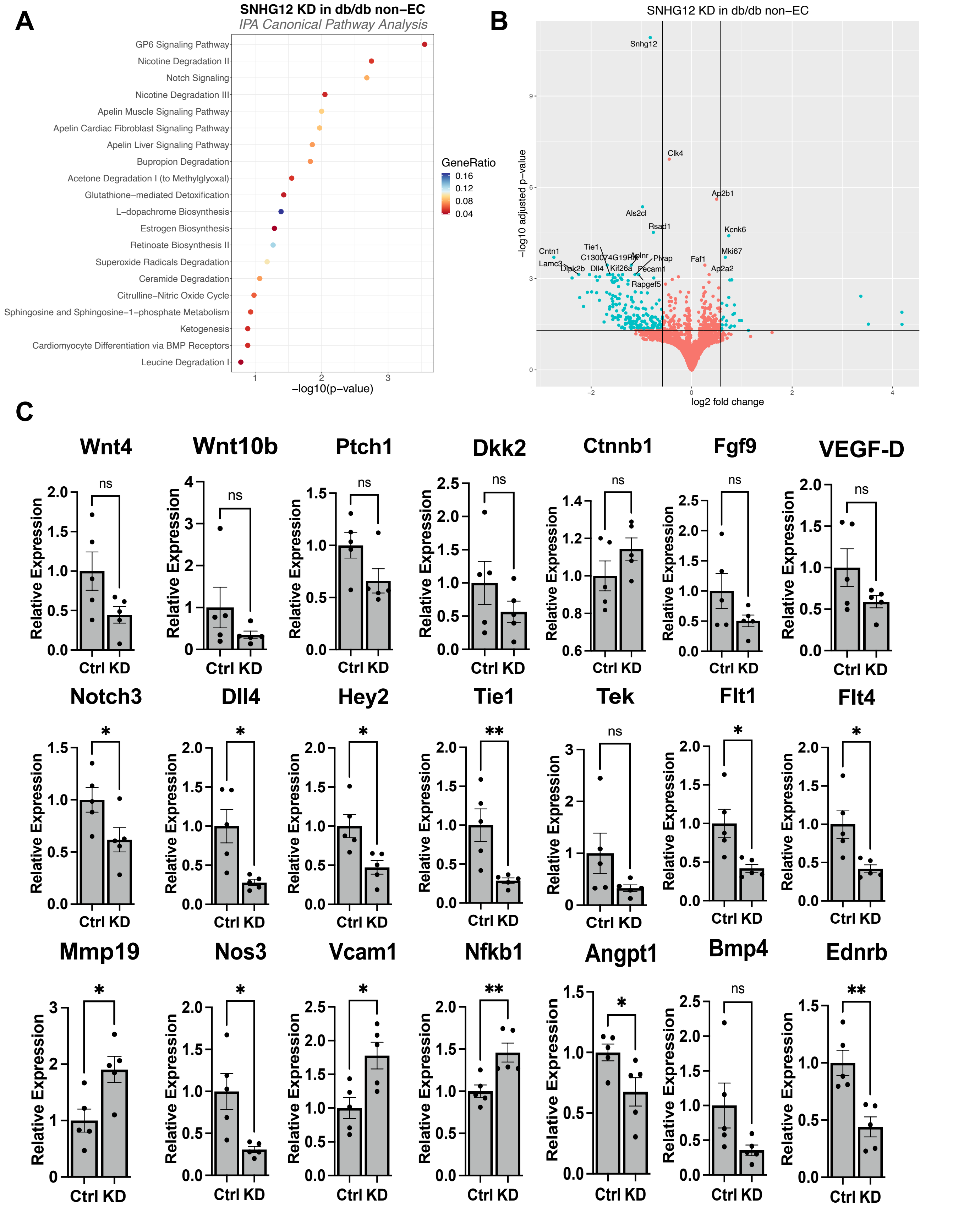

Figure 13S: RNA-seq analysis of Snhg12 knockdown in *db/db* non-ECs after FAL. (A) Ingenuity pathway analysis reveals various pathways that heavily represent Notch and BMP signaling differences and nitric oxide signaling pathway elements. **(B)** Volcano plot of most significant differentially expressed genes shows Tie1, Pecam1, Dll4 amongst top 20 most significant differentially expressed genes. **(C)** Selected genes plotted for relative expressionfrom RNA-seq data show a decreased Angpt1, Notch3, and Wnt pathway expression levels, and decreased eNOS (Nos3), with increased proinflammatory markers Vcam1, Nfkb1 and increased anti-angiogenic Mmp19. * P<0.05, ** P<0.01 using Student's t-test.
